# *N*^6^-methyladenosine-modified SRPK1 promotes aerobic glycolysis of lung adenocarcinoma via PKM splicing

**DOI:** 10.1186/s11658-024-00622-5

**Published:** 2024-08-02

**Authors:** Anqi Wang, Yuanyuan Zeng, Weijie Zhang, Jian Zhao, Lirong Gao, Jianjun Li, Jianjie Zhu, Zeyi Liu, Jian-an Huang

**Affiliations:** 1 Department of Pulmonary and Critical Care Medicine, The First Affiliated Hospital of Soochow University, Suzhou, 215006 China; 2 Institute of Respiratory Diseases, Soochow University, Suzhou, 215006 China; 3 Suzhou Key Laboratory for Respiratory Diseases, Suzhou, 215006 China

**Keywords:** m^6^A modification, Glycolysis, METTL3, SRPK1, Lung adenocarcinoma

## Abstract

**Background:**

The RNA *N*^6^-methyladenosine (m^6^A) modification has become an essential hotspot in epigenetic modulation. Serine–arginine protein kinase 1 (SRPK1) is associated with the pathogenesis of various cancers. However, the m^6^A modification of SRPK1 and its association with the mechanism of in lung adenocarcinoma (LUAD) remains unclear.

**Methods:**

Western blotting and polymerase chain reaction (PCR) analyses were carried out to identify gene and protein expression. m^6^A epitranscriptomic microarray was utilized to the assess m^6^A profile. Loss and gain-of-function assays were carried out elucidate the impact of METTL3 and SRPK1 on LUAD glycolysis and tumorigenesis. RNA immunoprecipitation (RIP), m^6^A RNA immunoprecipitation (MeRIP), and RNA stability tests were employed to elucidate the SRPK1’s METTL3-mediated m^6^A modification mechanism in LUAD. Metabolic quantification and co-immunoprecipitation assays were applied to investigate the molecular mechanism by which SRPK1 mediates LUAD metabolism.

**Results:**

The epitranscriptomic microarray assay revealed that SRPK1 could be hypermethylated and upregulated in LUAD. The main transmethylase METTL3 was upregulated and induced the aberrant high m^6^A levels of SRPK1. Mechanistically, SRPK1’s m^6^A sites were directly methylated by METTL3, which also stabilized SRPK1 in an IGF2BP2-dependent manner. Methylated SRPK1 subsequently promoted LUAD progression through enhancing glycolysis. Further metabolic quantification, co-immunoprecipitation and western blot assays revealed that SRPK1 interacts with hnRNPA1, an important modulator of PKM splicing, and thus facilitates glycolysis by upregulating PKM2 in LUAD. Nevertheless, METTL3 inhibitor STM2457 can reverse the above effects in vitro and in vivo by suppressing SRPK1 and glycolysis in LUAD.

**Conclusion:**

It was revealed that in LUAD, aberrantly expressed METTL3 upregulated SRPK1 levels via an m^6^A-IGF2BP2-dependent mechanism. METTL3-induced SRPK1 fostered LUAD cell proliferation by enhancing glycolysis, and the small-molecule inhibitor STM2457 of METTL3 could be an alternative novel therapeutic strategy for individuals with LUAD.

**Supplementary Information:**

The online version contains supplementary material available at 10.1186/s11658-024-00622-5.

## Background

Lung cancer is a major cause of death by cancer globally and is of two subtypes, small-cell and non-small-cell lung cancer (NSCLC) [1]. Lung adenocarcinoma (LUAD) accounts for a major portion of lung cancer cases [2]. Despite tremendous advances in treatment avenues, such as immunotherapy, targeted therapy and novel combined therapy, the prognosis of LUAD remains substandard [3]. Tumour recurrence and metastasis are still unresolved problems causing unfavourable outcomes, and the mechanisms underlying LUAD progression are still not fully elucidated [4]. Therefore, the identification and development of new molecular mechanisms and clinical approaches for LUAD remain urgent needs.

The modification of *N*^6^-methyladenosine (m^6^A) is the most frequent internal post-transcriptional messenger RNA modification and modulates a variety of eukaryotic biological processes [5]. This reversible alteration of m^6^A mainly comprises three components: RNA-binding proteins (readers), demethylases (erasers) and methyltransferases (writers) [6]. The methyltransferase complex (MTC) catalyses the m^6^A modification, while demethylases reverse the effect, and readers lead target RNA to different destinations. METTL3, as the core catalytic component of MTC, essentially associated with gene expression modulation via RNA translation, stability, degradation, and splicing [7, 8]. Recent literature has indicated that m^6^A methylation of mRNA induced by METTL3 may cause various mammalian diseases and tumours by modulating tissue development and cell differentiation and is a cause of tumorigenesis and tumour progression in different cancers [9–12]. For instance, METTL3 was observed to be crucial for lung cancer cell’s epithelial–mesenchymal transition mediated by TGF-β [13]. These studies proved that METTL3 is critically linked with tumorigenesis, whereas the biological significance and underlying mechanism of METTL3-mediated m^6^A modification in LUAD remain controversial due to the complexity of the m^6^A modification system.

Serine–arginine protein kinase 1 (SRPK1) is a protein kinase that phosphorylates serine/arginine-rich splicing factors (SRSFs). SRPK1 participates in various biological processes, including RNA maturation, alternative splicing, translation regulation and genomic stability, by interacting with RNA splicing factors [14–16]. SRPK1 and its downstream targets have also been shown to be involved in tumour progression, with increased expression identified in different cancers, such as breast, prostate and lung cancers [17–19]. Recent LUAD research has revealed that SRPK1 stimulated a stem cell-like phenotype by Wnt/β-catenin pathway activation [17]. Other studies have shown that targeting SRPK1 has antitumour effects, making SRPK1 a promising candidate for targeted therapies [20, 21]. Although much evidence has verified the importance of SRPK1 in tumorigenesis, no study to date has revealed the epigenetic regulatory mechanism of aberrant SRPK1 expression and the activity of SRPK1 in the metabolic reprogramming of tumour cells. This research discovered that METTL3-induced m^6^A modification modulated SRPK1 levels. More importantly, we found that methylated SRPK1 stimulated LUAD progression by enhancing glycolysis.

Growing evidence has demonstrated that metabolic reprogramming is a hallmark of cancer cells. In contrast to normal cells, tumour cells acquire most of their energy from glycolysis and not from the mitochondrial oxidative phosphorylation (OXPHOS), even during abundant oxygen, a process called the Warburg effect or aerobic glycolysis [22–24]. It has been indicated that aerobic glycolysis is linked with cell proliferation and survival by enhancing glucose consumption and lactate production in cancer cells [25]. Although less energy is produced by glycolysis than by mitochondrial oxidation, glycolysis is still preferred by tumour cells because it fulfils the biosynthetic demands associated with infinite malignant proliferation [26]. But the molecular basis for glycolysis and its relationship with m^6^A modification in LUAD still need further exploration. Here, we revealed that METTL3-methylated SRPK1 could enhance glycolysis by binding with its downstream RNA splicing factor. Heterogeneous nuclear ribonucleoprotein A1 (hnRNPA1) is a critical RNA-binding protein in regulating alternative splicing of *PKM* pre-mRNA which leads to a decrease in the PKM1/PKM2 ratio [27, 28]. Between the two isoforms of crucial glycolytic enzyme pyruvate kinase, in glycolysis PKM2 act as the rate-limiting enzyme, while PKM1 promotes OXPHOS [29, 30]. Thus, the SRPK1 interacting with hnRNPA1 could largely affect the metabolism of tumour cells by interfering with PKM splicing.

Different from the mechanism reported before, we elucidated the crucial function of METTL3-mediated SRPK1 in the glycolytic metabolism of LUAD. This research revealed that METTL3-mediated m^6^A modification promoted SRPK1 expression in an IGF2BP2-dependent manner, and upregulated SRPK1 promotes glycolysis in LUAD through hnRNPA1-mediated alternative PKM splicing. Our findings also provided more theoretical basis for clinical application of METTL3 inhibitor STM2457.

## Methods

### Tissue samples

A total of 41 paired fresh lung adenocarcinoma tissues and matched adjacent nontumor samples were collected from the First Affiliated Hospital of Soochow University between 2019 and 2022. The sample inclusion criteria were: (1) The samples were diagnosed LUAD based on their pathological and histological feature according to the Revised International System for Staging Lung Cancer. (2) The patient did not receive chemotherapy, radiotherapy, immunotherapy, and target therapy before surgery. (3) All the patients have signed informed consent, and relevant clinicopathological data are collected with the patient's consent. The sample exclusion criteria were: (1) Pathological examination did not confirm the diagnosis of LUAD. (2) The patient received preoperative radiotherapy and chemotherapy, immunotherapy, targeted therapy or other adjuvant therapy. (3) The patient did not consent to the collection of samples and clinicopathological data. Upon excision, the tissue samples were immediately frozen, preserved in an RNA stabilization solution, and stored at −80 ℃. The study was authorized by the Ethics Committee of the First Affiliated Hospital of Soochow University. Detailed information on the participants is presented in Table S1.

### Cell lines and cell culture

Human lung cancer cell types H1299 (CL-0165), H1650 (CL-0166), PC-9 (CL-0668), H1975 (CL-0298), HCC827 (CL-0094), A549 (CL-0016) (lung adenocarcinoma cell lines), H226 (CL-0396) (lung squamous carcinoma cell line), HEK293T (human embryo kidney cell), and BEAS-2B (human bronchial epithelial cell line) were acquired from the Procell Life Science and Technology Co. Ltd. (Wuhan, China). Short tandem repeat (STR) profiling was performed to authenticate all cell lines. Cells were cultivated in RPMI-1640 or DMEM media (Procell, Wuhan, China) augmented with 10% FBS (Gibco, CA, USA) and penicillin/streptomycin (100 mg/ml, Beyotime Biotechnology, Shanghai, China) at 37 ℃ in a 5% CO_2_ humid incubator.

### Transient transfection

Pre-designed sequences of short interfering RNA (siRNA) targeting various coding regions of SRPK1, METTL3, IGF2BP1, IGF2BP2, IGF2BP3, YTHDF1, YTHDF2, and hnRNPA1 were prepared by GenePharma (Suzhou, China). Table S2 enlists the target sequences. Scrambled siRNA was utilized as a negative control. The cells were then transiently transfected with siRNA by Lipofectamine 2000 (Invitrogen, CA, USA). We harvested the cell for subsequent experiments 72 h after.

### Establishment of stable cell lines

METTL3 and SRPK1 overexpression lentiviruses and control lentiviruses were acquired from GeneChem Corporation (Shanghai, China). The lentiviruses were infected in the cells per the manufacturer’s guide. Then to establish a stable cell line for the subsequent analyses, cell selection was performed with 1 µg/ml puromycin (Sigma‒Aldrich, St Louis, MO, USA).

### Total RNA extraction and real-time PCR

RNA was isolated, cDNA was prepared and real-time quantitative reverse transcription polymerase chain reaction (qRT–PCR) was carried out as described in the literature [31]. Table S3 enlists the primers utilized. The relative expression levels were quantified via the ΔΔCt method. β-Actin was employed as an endogenous control.

### Western blotting

This assay was performed per previous studies [31]. The utilized antibodies included anti-METTL3 (ab195352, Abcam, London, UK), anti-SRPK1 (sc-100443, Santa Cruz, CA, USA), anti-IGF2BP2 (11601-1-AP), anti-PKM1 (19987-1-AP), anti-PKM2 (15822-1-AP), anti-PCNA (60097-1-Ig), DYKDDDDK tag (66008-4-Ig), anti-PKM (25659-1-AP), anti-HK1 (19662-1-AP), anti-HK2 (22029-1-AP), anti-LDHA (19987-1-AP), anti-PFKFB3 (13763-1-AP), anti-hnRNPA1 (15821-1-AP, Proteintech, IL, USA), and anti-β-Actin (CW0096M, Cowin Bio, Jiangsu, China) antibodies. Furthermore, goat anti-mouse IgG, HRP-conjugated (CW0102) and anti-rabbit IgG, HRP-conjugated (CW0103, Cowin, Jiangsu, China) antibodies were utilized as secondary antibodies. The electrochemiluminescence reagent (Thermo Fisher Scientific, MA, USA) was used for bands development, imaged using a ChemiDoc XRS + (Bio-Rad, CA, USA), and lastly quantified with ImageJ software (National Institutes of Health, MD, USA).

### Cell counting kit-8 (CCK-8) assay

CCK-8 assay was carried out as previously described [31]. For assessing, cell viability, CCK-8 (Boster, Wuhan, China) was utilized per the kit’s instructions.

### 5-Ethynyl-2′-deoxyuridine (EdU) assay

The EdU assay (C10310-1, Ribobio, Guangdong, China) was carried out using the guide provided by the manufacturer as mentioned in previous study [32].

### Cell invasion and migration assays

Cell invasion and migration assessment was carried out based on the previous studies [31]. For the invasion assay, Matrigel matrix (Corning, NY, USA) was used to coat the inserts. The cells were then imaged and counted under a microscope.

### M^6^A RNA methylation quantification

According to the previously described method, total RNA was isolated [31]. Then, the m^6^A RNA methylation levels were quantified using the acquired RNA via the EpiQuik m^6^A RNA Methylation Quantitation Kit (Colorimetric) (#P-9005, Epigentek, NY, USA) per the kit’s instructions. The m^6^A levels were measured by assessing the absorbance at 450 nm.

### M^6^A–mRNA and long non-coding RNA (lncRNA) epitranscriptomic microarray analysis

Whole RNA from 12 clinical samples (6 pairs of LUAD and adjacent non-cancerous tissue) was extracted. The protocol of Arraystar Human mRNA and lncRNA Epitranscriptomic Microarray (8 × 60,000, Arraystar) was utilized for the sample preparation and microarray hybridization. Agilent Scanner G2505C was utilized for scanning the arrays. Agilent Feature Extraction software (version 11.0.1.1) was employed to evaluate the obtained array images. Microarray experiments and data assessment were conducted by KangChen Biotech (Shanghai, China).

### RNA immunoprecipitation (RIP)

Magna RIP™ RNA-Binding Protein Immunoprecipitation Kit (No. 17-700, Merck Millipore, MA, USA) was employed for RIP assay, according to the kit’s guide. Briefly, using the kit’s RIPA buffer, the cells were lysed and collected and incubated with RIP buffer comprising magnetic beads linked with the relevant antibody or control normal IgG. For sample digestion, proteinase K was employed to isolate the immunoprecipitated RNA, which was then subjected to qRT‒PCR to identify the presence of the binding targets.

### M^6^A RIP (MeRIP)

For MeRIP analysis, a Magna MeRIP™ m^6^A Kit (No. 17-10499, Merck Millipore, MA, USA) was employed, per the kit’s instructions as previously mentioned [33]. The m^6^A-containing mRNA enrichment was assessed by qRT‒PCR and normalized to the input. The primers utilized for MeRIP PCR are provided in Table S4.

### RNA stability

Actinomycin D (Act-D, 5 μg/ml) (cat. no. S8964, Selleck, TX, USA) was added to the LUAD cells for RNA stability. The cells were collected after incubation at the indicated times for RNA isolation via TRIzol reagent, and then reverse transcription was carried out to measure the remaining mRNA using qRT‒PCR.

### Immunofluorescence staining

Immunofluorescent staining was performed as described previously [31]. The stained cells images were acquired via a confocal microscope (ZEISS, BW, Germany) under standardized conditions.

### Luciferase assay

Fragments of SRPK1–3′ untranslated region (UTR) comprising the wild-type and mutant m^6^A motifs (C instead of m^6^A) were prepared at Azenta Life Science (Shanghai, China). The fragments of mutant and wild-type SRPK1–3′ UTR were inserted into the psiCHECK2 luciferase vector. Cells were propagated in 24-well plates and cotransfected with 500 ng wild-type/mutant luciferase reporter and 0.5 μg control/SRPK1-overexpressing plasmid. The relative luciferase activity, cell lysates were collected and Dual-Luciferase Reporter Assay kit (Promega, WI, USA) was utilized 24 h later.

### Quantification of energy metabolites by liquid chromatography tandem mass spectrometry (LC–MS/MS)

MetWare (http://www.metware.cn/) were utilized to detect all the metabolites based on the AB Sciex QTRAP® 6500 LC–MS/MS platform. Then the pathways with substantially modulated metabolites mapped were imported in metabolite set enrichment analysis (MSEA), and their significance was assessed via the *p* values of the hypergeometric test.

### Extracellular acidification rate (ECAR)

The ECAR was assessed with the help of a Seahorse Glycolysis Stress Test Kit (103017) on a Seahorse XFp Analyser (Agilent Technologies, CA, USA), per the kit’s guide. Briefly, 10,000 cells per well were propagated in a Seahorse XFp cell culture microtiter plate for 24 h and then used for ECAR measurement. After the baseline assessment, 10 mM of glucose, 1 μM of oligomycin (oxidative phosphorylation inhibitor), and the 50 mM of 2-DG (glycolysis inhibitor) were sequentially added to each well at a specified timepoint. The data were analysed by Seahorse Wave software. The cells were counted again after the measurement. The results were normalized to the number of cells.

### Glucose uptake and lactate generation analysis

For the glucose uptake assay, cells with a density of 5 × 10^6^/ml were collected and resuspended in 1 ml of distilled water. The suspension underwent ultrasonication, 10 min of boiling, and then centrifuged at 25 ℃ and 8000*g* for 10 min. A glucose detection kit (#BC2500, Solarbio Science & Technology, Beijing, China) was then used per the kit’s protocols. The absorbance at 505 nm was measured.

For the lactate production assay, cells with a density of 5 × 10^6^/ml were collected and resuspended in extracting solution from the lactate detection kit (#BC2230, Solarbio Science & Technology, Beijing, China). The following steps were performed per the kit’s method. The absorbance was measured at 570 nm.

### Co‑immunoprecipitation

Co-immunoprecipitation was carried out as mentioned previously [31]. IgG-, hnRNPA1-, SRPK1-, or Flag-bound proteins were isolated via SDS-PAGE and then subjected to western blotting assay.

### In vivo xenograft model

BALB/c athymic nude mice (aged 4–6 weeks, female, with a weight of 16–20 g) were acquired from the Laboratory Animal Center of Suzhou Medical College, Soochow University and bred in pathogen-free environment. The first batch of 16 mice was randomly categorized into two cohorts (8 per group). For model establishment, control or METTL3-overexpressing A549 cells suspended in 100 μl 1640 RPMI medium with 50% Matrigel (serum-free) and injected subcutaneously into nude mice flanks. Tumour volumes (*V*) were identified by assessing the tumour length (*L*) and width (*W*) with a Vernier calliper via the following formula: *V* = *(L* × *W*^*2*^) × 0.5. The second batch of 18 mice was first randomly divided into two groups: the control (*n* = 6 mice) and the SRPK1-overexpressing (*n* = 12 mice), inoculated with control cells and SRPK1-overexpressing cells, respectively. At the tumour volume of 100–150 mm^3^, the overexpressing group was once again randomly divided into the vehicle group and STM2457 group. Then the mice were treated with vehicle or 50 mg/kg/day STM2457 (cat. no. S9870, Purity 99.95%, Selleck, TX, USA) via intraperitoneal injection until sacrifice. All in vivo analyses were carried out by following the Guide for the Care and Use of Experimental Animals Center of Soochow University.

### Statistical measurements

All assays were conducted independently in triplicate. GraphPad Prism 9.0 (GraphPad, CA, USA) was utilized for all the statistical assessments and the data are illustrated as the mean ± SD. The intergroup significant differences were elucidated by a nonpaired Student’s* t*-test. Whereas for the significant differences between more than two groups, one-way or two-way ANOVA was utilized. All statistical tests were two-tailed and *P* < *0.05* was deemed statistically significant.

## Results

### Aberrant METTL3 level promotes high *N*^6^-methyladenosine modification in LUAD

**Fig. 1:**
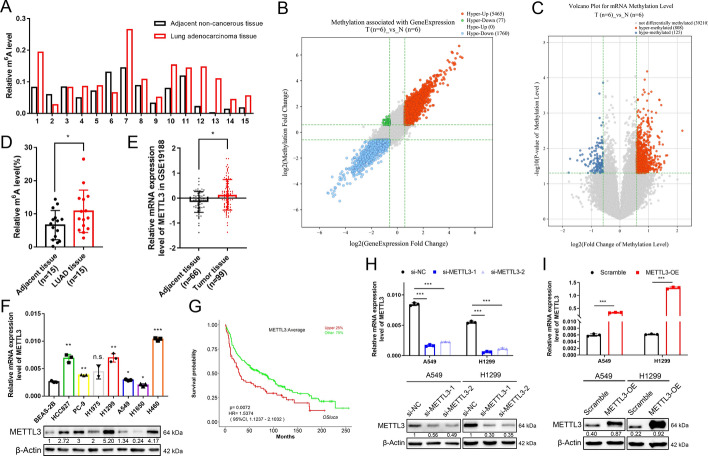
Aberrant METTL3 expression and high m^6^A level in LUAD. **A**, **D** Bar chart and dot histogram of m^6^A levels in 15 pairs of human lung adenocarcinoma and surrounding non-cancerous tissues. **B** Scatter plot of mRNAs with differentially methylated levels and differentially mRNA levels. The coloured dots suggest a more than 1.5-fold change in both methylation level and mRNA expression. **C** Volcano plot of mRNAs with significantly differentially methylated levels. The coloured dots suggest a methylation level of both *p-*value < *0.05* and fold change > 1.5. **E** Data from the GEO database (GSE19188) indicated that mRNA levels of METTL3 are substantially increased in lung cancer tissues compared with adjacent tissue samples. **F** Western blotting and qRT‒PCR assessments of METTL3 protein and mRNA levels in different lung cancer and a normal bronchial epithelial cell lines. **G** Kaplan–Meier overall survival curves of METTL3 by the OSluca database (http://bioinfo.henu.edu.cn/LUCA/LUCAList.jsp). **H**, **I** The protein and mRNA levels of METTL3 in METTL3 overexpressing and knockdown cells. *P < 0.05; ** *P *< 0.01; ***P < 0.001

To examine the abnormal *N*^6^-methyladenosine (m^6^A) modification in lung adenocarcinoma (LUAD), 15 paired samples of LUAD and adjacent non-cancerous tissue were taken from our tissue sample collection to perform m^6^A quantification analysis, and the data indicated that the overall m^6^A level was markedly higher in most LUAD samples (80%, 12/15) than in adjacent tissue samples (Fig. 1A and D). We further performed an epitranscriptomic microarray analysis in six other LUAD and paired non-tumour tissue samples to investigate the m^6^A modification of mRNA transcripts. An overview of the fold changes in both m^6^A modification and mRNA expression is shown in the scatter diagram (Fig. 1B), and most of the mRNAs (74.8%, 5465/7302) were both hypermethylated and overexpressed. The volcano map of methylation levels showed that there were almost seven times more hypermethylated genes than hypomethylated genes (808 versus 125) (Fig. 1C). These results revealed a relative hypermethylation trend in LUAD, thus leading us to wonder about the cause of such abnormal m^6^A levels.

Naturally, we think of the well-recognized transmethylase METTL3, which plays the main catalytic role in mRNA m^6^A modification. METTL3 was also found to be upregulated in lung cancer in previous studies. We examined data from Gene Expression Omnibus (GEO, GSE19188) and confirmed that METTL3 levels were higher in LUAD tissues (Fig. 1E). We also found that METTL3 mRNA and protein levels were higher in most lung cancer cells (6/7, HCC827, PC-9, H1975, H1299, A549, H460) than in normal bronchial epithelial cells (BEAS-2B) (Fig. 1F). Kaplan‒Meier analysis from the OSluca database (http://bioinfo.henu.edu.cn/LUCA/LUCAList.jsp) indicated that high METTL3 levels were associated with poor overall survival (Fig. 1G). These results indicated that METTL3 plays an oncogenic role in LUAD and that its aberrantly high expression level possibly induces hypermethylation in LUAD.

### The role of METTL3 in mediating LUAD cell proliferation and tumour growth

**Fig. 2:**
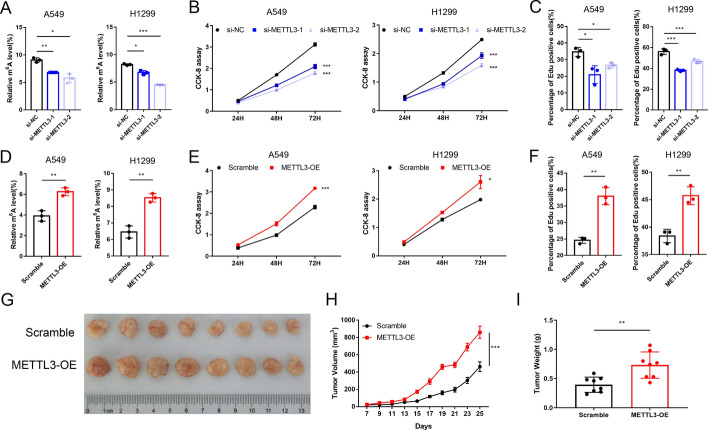
METTL3 regulates m^6^A modification and promotes tumour growth in LUAD. **A**, **D** The m^6^A level in METTL3 overexpressing and knockdown cells. **B**, **E** CCK-8 analysis of A549 and H1299 cell viability with METTL3 knockdown or overexpression. **C**, **F** Quantification bar chart of the EdU analysis of METTL3 overexpressing and knockdown A549 and H1299 cells. **G** Representative images of control and METTL3-overexpressing xenograft tumours. **H** Tumour volume of the control and METTL3 overexpression groups measured at day 7, 9, 11, 13, 15, 17, 19, 21, 23 and 25 after subcutaneous injection. **I** Tumour weight of the control and METTL3 overexpression groups after sacrifice. Data illustrated as the mean [± standard deviation (SD)] of three independent experiments. Two-way analysis of variance (ANOVA) and unpaired *t*-tests were carried out to verify the statistical significance. *P < 0.05; **P < 0.01; ***P < 0.001

To further validate the role of METTL3 in LUAD oncogenesis, we altered the expression of METTL3 in LUAD cell lines (A549 and H1299). The mRNA and protein levels of METTL3 were significantly reduced after transfection with two small-interfering RNAs (siRNAs) against METTL3 (Fig. 1H), and stable METTL3-overexpressing cell lines were established with the corresponding lentivirus (Fig. 1I). The m^6^A quantification assay was performed again to confirm the main transmethylase role of METTL3 in LUAD. The m^6^A level of si-METTL3 cells was decreased, while that of METTL3-overexpressing cells was in turn increased (Fig. 2A and D). CCK-8 assays were performed to assess the effect of METTL3 on cell proliferation. METTL3 knockdown inhibited cell proliferation, while METTL3 overexpression significantly promoted cell growth (Fig. 2B and E). EdU assays further verified the above results. The percentage of EdU-positive cells decreased after METTL3 interference, while an increased percentage of EdU-positive cells was observed in METTL3-overexpressing cells (Fig. 2C and F, Figure. S1). In addition, we found that the xenograft tumours formed by METTL3-overexpressing cells were both larger in size and heavier in size than those formed by control cells as well (Fig. 2G–I). Collectively, these results showed that METTL3 promoted LUAD tumour growth in vitro and in vivo.

### Post-transcriptional sequencing identifies potential targets of METTL3 in LUAD

**Fig. 3:**
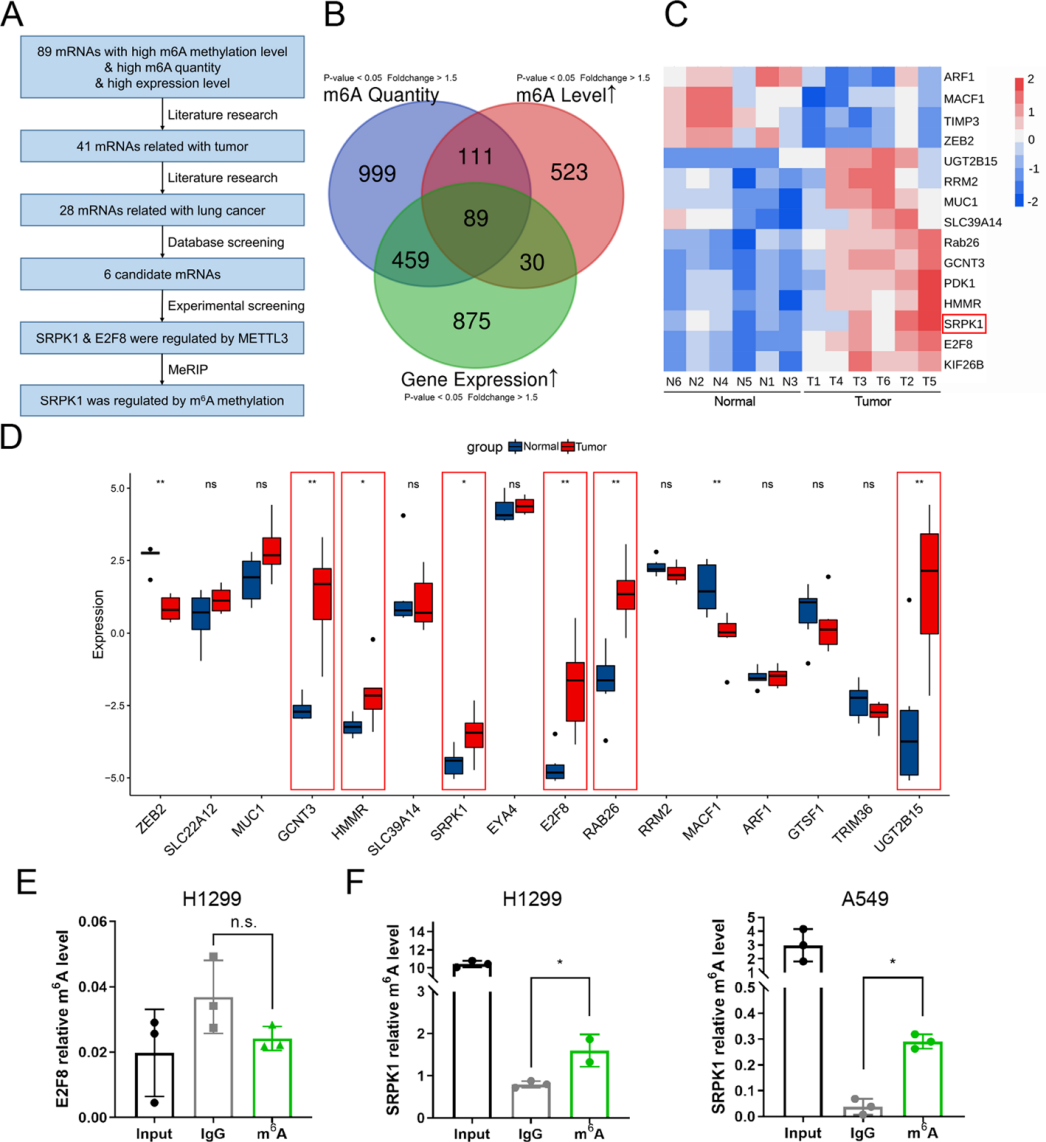
SRPK1 is the potential target of METTL3 in LUAD. **A** Flowchart for screening potential targets of METTL3. **B** Venn diagram of genes with differential m^6^A quantity, m^6^A level, and mRNA expression. **C** Heatmap of m^6^A levels of related genes in the microarray. **D** Bar chart of related genes expression in the TCGA database. **E** The H1299 cell’s m^6^A levels of E2F8 were determined by MeRIP–qPCR. **F** The A549 and H1299 cell’s m^6^A level of SRPK1 was assessed by MeRIP–qPCR. *P < 0.05; **P < 0.01

**Fig. 4:**
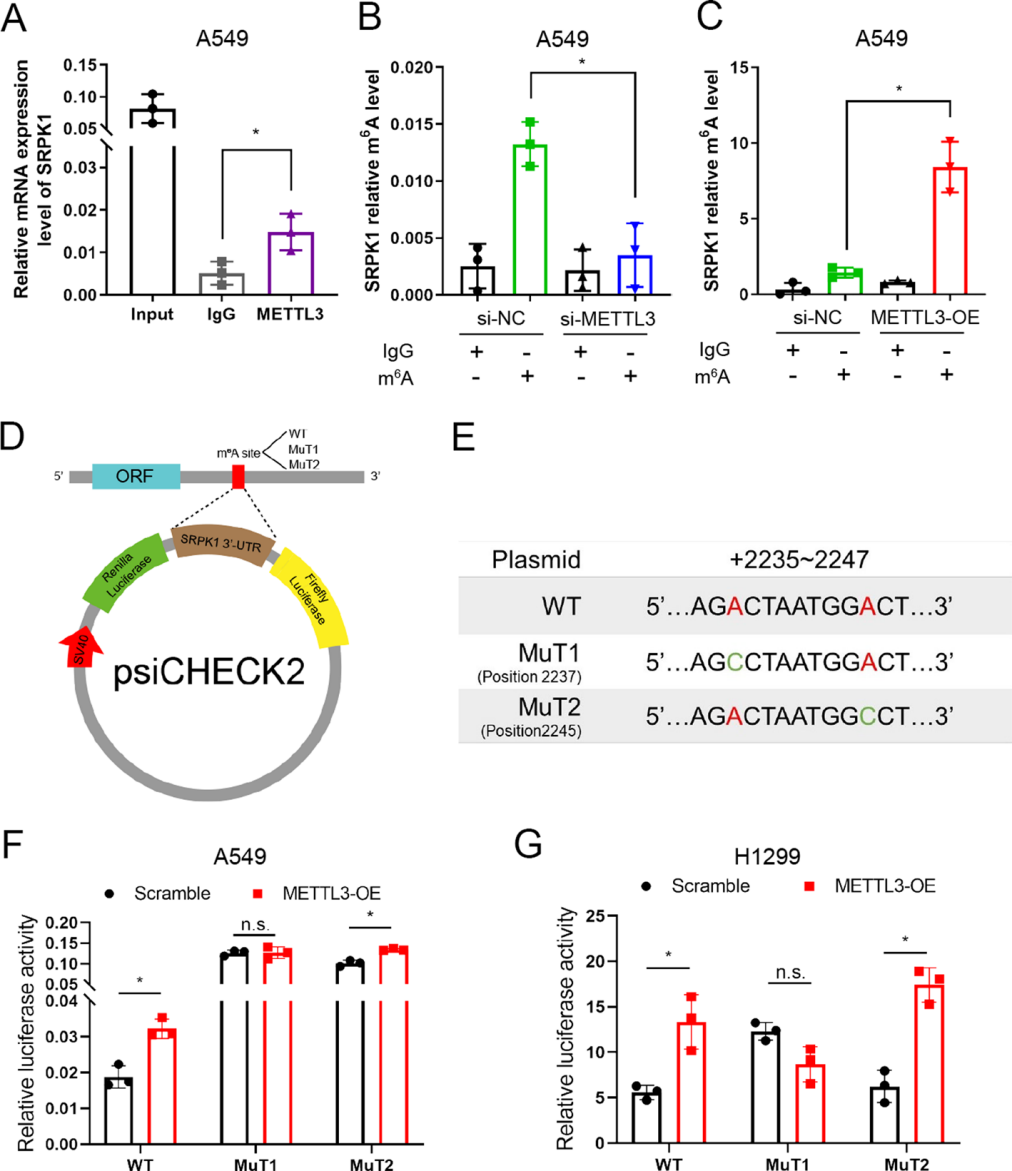
METTL3 mediates m^6^A modification of SRPK1 in LUAD. **A** The interaction of METTL3 with SRPK1 mRNA was analysed by RIP. **B**, **C** The m^6^A level of m^6^A in SRPK1 in A549 cells with METTL3 knockdown or overexpression was analysed by MeRIP–qPCR. **D**, **E** Putative binding sites of METTL3 in the 3′-UTR sequences of SRPK1 and the sequence of two mutant plasmids. **F**, **G** Luciferase activities were detected in A549 and H1299 cells. Data are illustrated as the mean (± SD) of three separate experiments. An unpaired *t*-test was performed to validate the statistical significance. *P < 0.05

Nevertheless, the underlying mechanism by which METTL3 promotes tumour growth in LUAD remained unclear. We analysed the results of the m^6^A microarray mentioned above to investigate the potential targets of METTL3, as shown in the flowchart (Fig. 3A). We drew a Venn diagram to find the intersection of 89 genes with hypermethylation level, upregulated quantity and high gene expression (fold change > 1.5, *P* < 0.05) (Fig. 3B). A heatmap of the methylation levels of representative genes is also shown (Fig. 3C). By preliminary screening of existing literature, we narrowed the list down to 28 candidate genes relevant to lung cancer that might be methylation targets of METTL3. Subsequently, we ruled out the genes with low expression in LUAD by The Cancer Genome Atlas (TCGA) database (Fig. 3D) and the genes unchanged in METTL3-knockdown cells (Fig. S2A). We then narrowed the list down to two candidates, SRPK1 and E2F8, when MeRIP results revealed that SRPK1 was m^6^A modified, while E2F8 was not (Fig. 3E and F). RIP assay demonstrated that METTL3 transmethylase interacted with *SRPK1* mRNA (Fig. 4A). We further performed MeRIP in METTL3-inhibited cells and METTL3-overexpressing cells. The m^6^A modification of *SRPK1* mRNA was significantly reduced after METTL3 was knocked down, and the opposite was observed in METTL3-overexpressing cells (Fig. 4B and C).

To elucidate the specific m^6^A site on *SRPK1* mRNA, the two most likely m^6^A methylation sites in *SRPK1* were predicted by the SRAMP database (http://www.cuilab.cn/sramp) [34] (Fig. S2B). We then cloned the wild-type (WT) SRPK1 3′ UTR (approximately +2235 to +2247 relative to the transcription start site; TSS), mutant 1 (MuT1) 3′ UTR (+2237, AGAC to AGCC), and mutant 2 (MuT2) 3′ UTR (+2245, GGAC to GGCC) downstream of the Renilla luciferase encoding region in the psiCHECK2 plasmid (Fig. 4D and E). The dual luciferase results suggested that site +2237 (MuT1) was the specific site for METTL3 to install the m^6^A modification on SRPK1 (Fig. 4F and G). These results confirmed that METTL3 mediates the m^6^A modification at site +2237 of *SRPK1*, and thus, we identified SRPK1 as a critical methylation target of METTL3 in LUAD.

### METTL3 regulates the expression of SRPK1 in an IGF2BP2-m^6^A-dependent manner

**Fig. 5:**
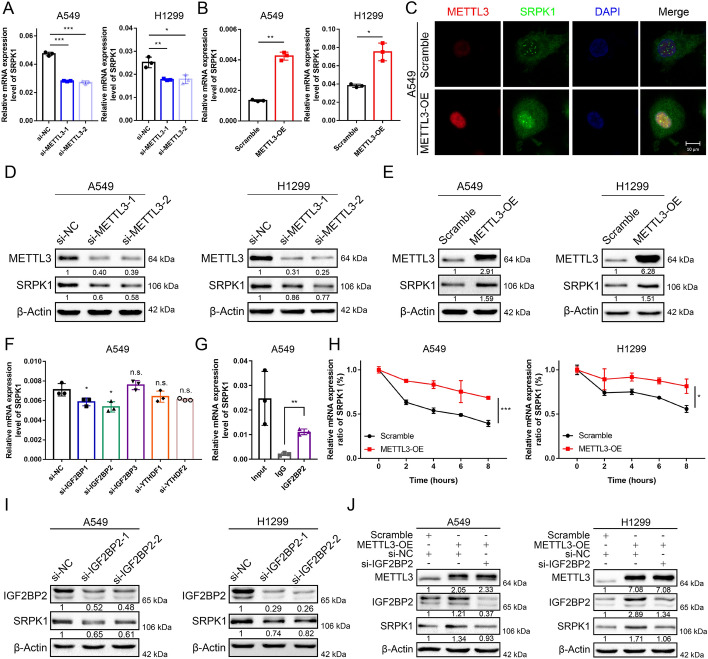
METTL3 promotes SRPK1 in an IGF2BP2–m^6^A-dependent manner. **A** SRPK1 mRNA levels in METTL3-knockdown H1299 and A549 cells by qRT‒PCR. **B** SRPK1 mRNA levels in METTL3-overexpressing H1299 and A549 cells by qRT‒PCR. **C** Immunofluorescence staining of METTL3 and SRPK1 co-expression in METTL3-overexpressing cells compared to control cells in A549 cells. **D** SRPK1 protein levels in METTL3-knockdown H1299 and A549 cells by western blot. **E** SRPK1 protein levels in METTL3-overexpressing H1299 and A549 cells by Western blot. **F** The SRPK1 mRNA level after interference with different representative m^6^A readers in A549 cells. **G** The interaction of IGF2BP2 with SRPK1 mRNA was analysed by RIP in A549 cells. **H** The stability of SRPK1 mRNA was detected in METTL-overexpressing cells treated with actinomycin D. **I** Western blot assay of SRPK1 and IGF2BP2 in IGF2BP2-knockdown A549 and H1299 cells. **J** Western blot analysis of METTL3, SRPK1 and IGF2BP2 protein levels in METTL3-overexpressing cells with or without knockdown of IGF2BP2. Data are illustrated as the mean (± SD) of three separate experiments. An unpaired *t*-test was performed to validate the statistical significance. *P < 0.05; **P < 0.01; ***P < 0.001

As we have discovered that SRPK1 is specifically methylated by METTL3, we continue to explore the impact of m^6^A modification on SRPK1 and the underlying mechanism. We found that the mRNA and protein levels of SRPK1 were positively regulated by METTL3 in A549 and H1299 cells (Fig. 5A, B, D, E). Immunofluorescence assays also revealed that METTL3 overexpression induced a corresponding increase in SRPK1 expression (Fig. 5C). Because the impact of m^6^A modification on target RNA is decided by the RNA-binding “m^6^A reader” [35, 36], we then screened the main m^6^A readers by qRT‒PCR and found out that interference with IGF2BP2-mediated SRPK1 expression most significant (Fig. 5F). RIP assays confirmed that IGF2BP2 directly interacted with *SRPK1* mRNA (Fig. 5G). IGF2BP2 was shown to stabilize mRNA in a previous study, and our RNA stability analysis also found that METTL3 overexpression enhanced the stability of SRPK1 (Fig. 5H). These results proved that *SRPK1* mRNA is recognized by IGF2BP2 after methylated by METTL3 and that its stability increased as its methylation level increases. Western blotting showed that SRPK1 was downregulated when IGF2BP2 was inhibited (Fig. 5I), and METTL3-mediated SRPK1 overexpression could be reversed by IGF2BP2 (Fig. 5J), showing that the changes in *SRPK1* RNA continued to induce changes on protein level. Collectively, we found that METTL3 regulates SRPK1 expression by inducing m^6^A modification at site +2237 and increasing its stability in an IGF2BP2-dependent manner.

### SRPK1 promotes cell proliferation in LUAD

We then investigated the oncogenic role of SRPK1 in LUAD. Notably, SRPK1 was also upregulated in LUAD tissues in the GEO database (GSE19188), GEPIA database and The Human Protein Atlas database (https://www.proteinatlas.org/) (Fig. S3A–S3C). A higher level of SRPK1 led to a shorter overall survival of LUAD patients (Fig. S3D). We also altered the expression of SRPK1 in A549 and H1299 cell lines (Fig. S3E and S3F). CCK-8 assays were performed to assess the effect of SRPK1 on cell proliferation. SRPK1 knockdown inhibited cell proliferation while overexpressing SRPK1 significantly promoted cell growth (Fig. S3G and S3H). EdU assays further verified the above results. The percentage of EdU-positive cells decreased after SRPK1 interference, while an increased percentage of EdU-positive cells was observed in SRPK1-overexpressing cells (Fig. S3I and S3J). Collectively, these results showed that SRPK1 also played an oncogenic role in LUAD.

### Methylated SRPK1 enhances glycolytic flux by regulating PKM1/2

**Fig. 6:**
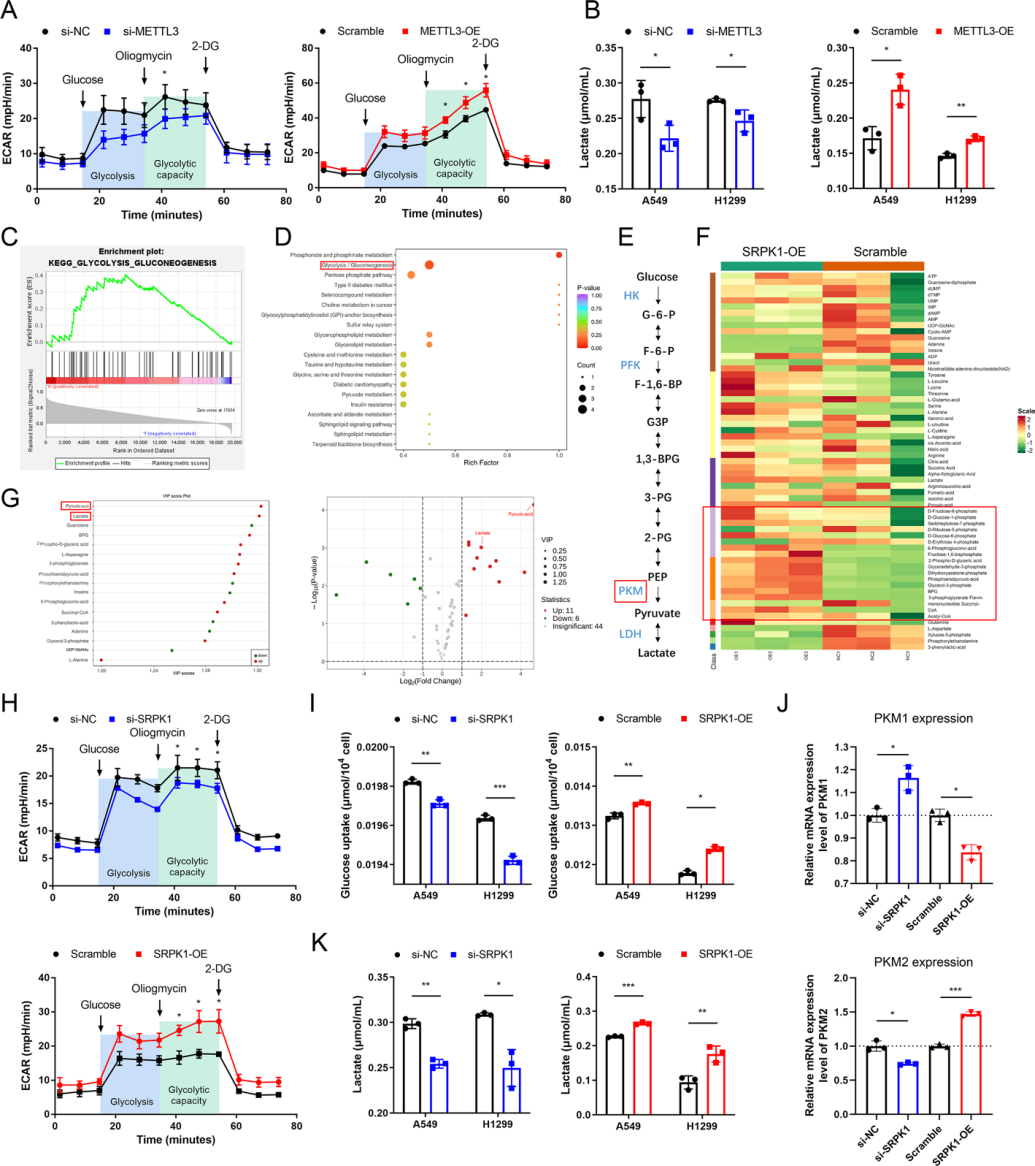
SRPK1 promotes glycolysis by regulating PKM1/2 expression. **A** ECAR was detected in METTL3-knockdown and METTL3-overexpressing cells. **B** Lactate production was measured in METTL3-knockdown and METTL3-overexpressing cells. **C** Kyoto Encyclopedia of Genes and Genomes (KEGG) pathway analysis of SRPK1 using TCGA data was conducted. **D** Pathway analysis using data from the quantification array of energy metabolism. **E** A concise diagram of glycolysis in cancer cells. **F** Heatmap of metabolites differentially expressed between control and SRPK1-overexpressing cells from energy metabolism quantification array data. **G** Scatter and volcano map of differentially expressed compounds. **H** ECAR was detected in SRPK1-knockdown and SRPK1-overexpressing cells. **I** Glucose uptake was measured in SRPK1-knockdown and overexpressing cells. **J** The mRNA levels of PKM1 and PKM2 in SRPK1-knockdown and SRPK1-overexpressing cells. **K** Lactate production was measured in SRPK1-knockdown and overexpressing cells. Data are illustrated as the mean (± SD) of three separate experiments. An unpaired *t*-test was performed to validate the statistical significance. *P < 0.05; **P < 0.01; ***P < 0.001

Now that we had found that METTL3 targets SRPK1 and promotes tumour growth in LUAD, the mechanism by which SRPK1 influenced LUAD progression was further explored. METTL3 was reported to promote glycolysis in lung cancer in previous studies, and we confirmed that changes in METTL3 expression regulated glucose uptake, lactate production, and the extracellular acidification rate (ECAR) in LUAD (Fig. 6A, B, and Fig. S4A). However, to our knowledge, no study has explained exactly how METTL3 regulates glycolysis in LUAD thus far. We therefore analysed the function of SRPK1 and investigated whether SRPK1 is the key by which METTL3 regulates glycolysis. Gene set enrichment analysis (GSEA) was performed using TCGA data, and the results showed that genes involved in glycolysis were enriched in the samples with high SRPK1 expression (Fig. 6C); thus, the different intermediate products of energy metabolism between control and SRPK1-overexpressing cells were analysed using LC‒MS/MS. The results showed that glycolysis-related metabolites were significantly higher in SRPK1-overexpressing cells, and pathway analysis also indicated that the differentially expressed metabolites were highly associated with glycolysis (Fig. 6D–F, Table S5). Moreover, the two important downstream metabolites of glycolysis, lactate and pyruvate, were most significantly differentially expressed (Fig. 6G). In the cellular functional experiments, ECAR analysis confirmed that SRPK1 knockdown reduced the glycolytic capacity and that SRPK1 overexpression promoted it (Fig. 6H). Glucose uptake and lactate secretion were both significantly decreased after SRPK1 knockdown, while SRPK1 overexpression facilitated glucose uptake and lactate production (Fig. 6I and K). Since pyruvic acid and lactate were most evidently increased in SRPK1-overexpressing cells, we next investigated the mRNA expression of critical upstream kinase of pyruvic acid, pyruvate kinase 1/2 (PKM1/2), in SRPK1-overexpressing or downregulated cells (Fig. 6J). The results revealed that the alteration of SRPK1 expression was positively related to PKM2 expression and negatively regulated PKM1 expression, which was in accordance with the result that SRPK1 promoted glycolysis. Meanwhile, the level of other major glycolysis-related genes (HK1, HK2, LDHA, PFKFB3) showed no significant differences in SRPK1-knockdown cell lines (Fig. S4B). Taken together, these findings demonstrated that SRPK1 enhanced the Warburg effect by regulating the rate-limiting enzyme PKM1/2 and hence promoting tumour growth in LUAD.

### The METTL3/SRPK1 axis promotes LUAD cell proliferation by activating glycolysis

**Fig. 7:**
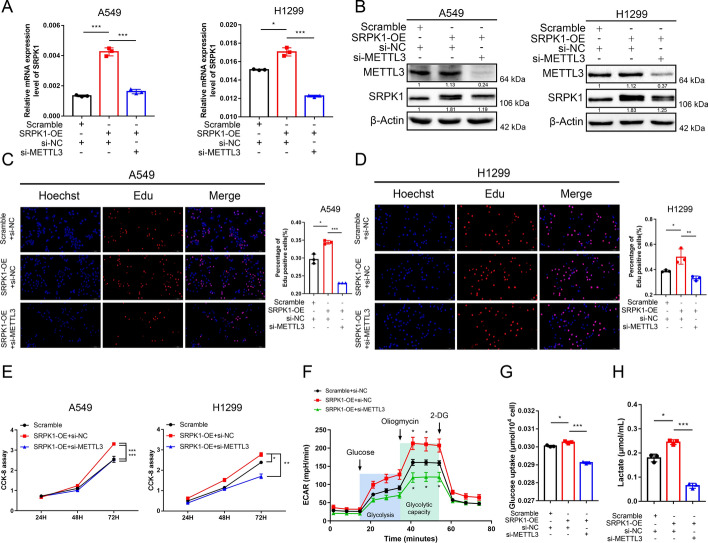
METTL3/SRPK1 axis promotes LUAD growth by enhancing glycolysis.** A** METTL3 knockdown inhibits SRPK1 overexpression on RNA level. **B** METTL3 knockdown inhibits SRPK1 overexpression on protein level. **C**, **D** METTL3 knockdown inhibits SRPK1-mediated cell proliferation, as detected by EdU assays. **E** METTL3 knockdown inhibits SRPK1-mediated cell proliferation, as detected by CCK-8 assays. **F** METTL3 knockdown inhibits the SRPK1-induced high ECAR curve. **G**, **H** METTL3 knockdown inhibits SRPK1-induced high lactate production and glucose uptake. Data are illustrated as the mean (± SD) of three separate experiments. An unpaired *t*-test was performed to validate the statistical significance. *P < 0.05; **P < 0.01; ***P < 0.001

To further elucidate the role of the METTL3/SRPK1 axis in the Warburg effect in LUAD, rescue assays were performed in A549 and H1299 cells. We first confirmed that knockdown of METTL3 could suppress SRPK1 expression in SRPK1-overexpressing cells (Fig. 7A and 7B). CCK-8 assays and EdU assays illustrated that SRPK1 overexpression enhanced cell proliferation, while METTL3 knockdown reversed these phenotypes (Fig. 7C–E). We then performed ECAR analysis, glucose uptake analysis and lactate production analysis to investigate the impact of the aforementioned axis on glycolytic metabolism (Fig. 7F–H). The results showed that knockdown of METTL3 could reverse the enhanced glycolytic phenotypes induced by SRPK1 overexpression, which further confirmed the regulatory relationship between METTL3 and SRPK1. In conclusion, METTL3-methylated SRPK1 expression accelerated glycolytic metabolism in LUAD cells and thus promoted the cell proliferation of LUAD.

### SRPK1 mediates PKM1/2 splicing by interacting with hnRNPA1

**Fig. 8:**
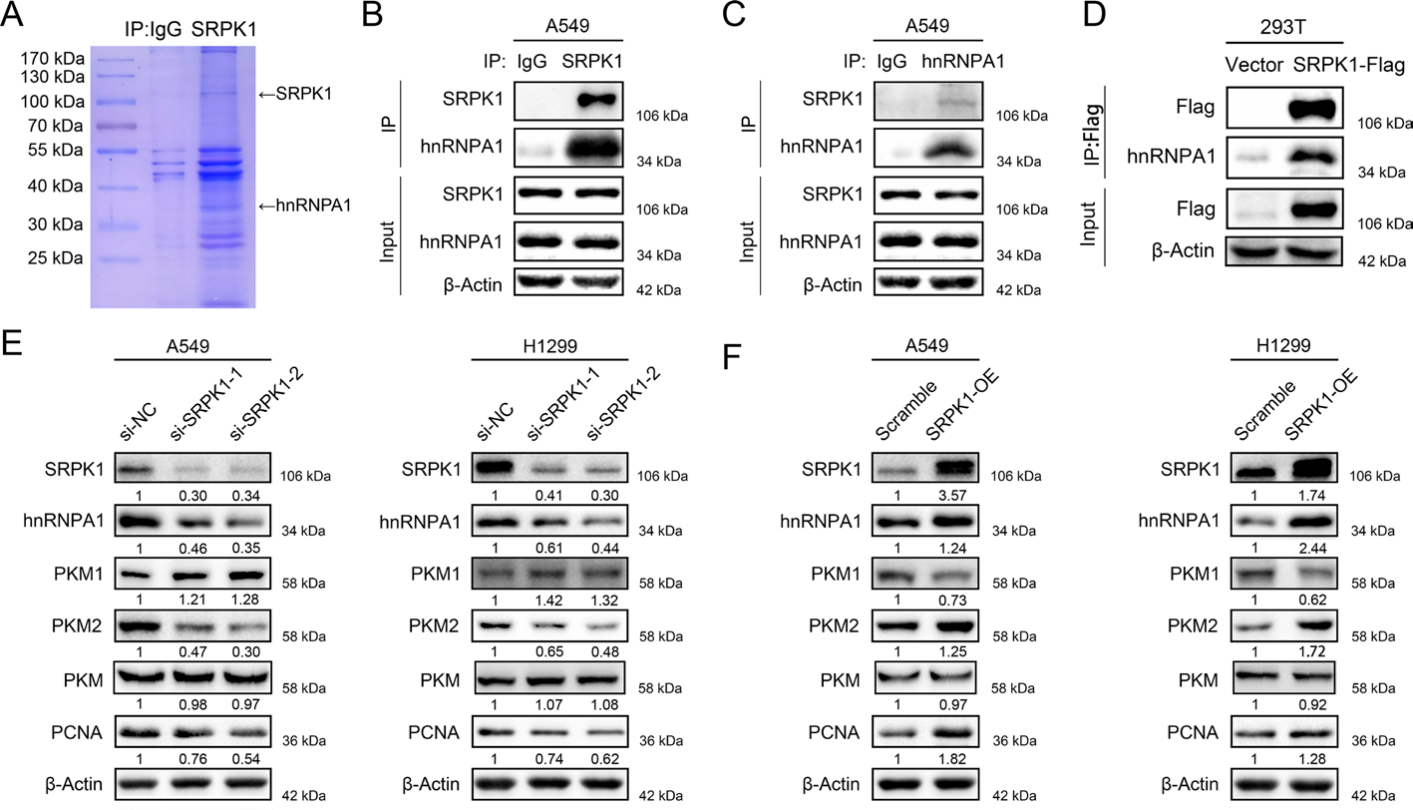
SRPK1 mediates PKM1/2 splicing by interacting with hnRNPA1. **A** Coomassie blue staining of SRPK1-immunoprecipitation (IP) cell lysates. **B** Immunoprecipitation of the hnRNPA1 protein by an anti-SRPK1 antibody in A549 cells. IgG was mediated as a negative control. **C** Immunoprecipitation of the SRPK1 protein by an anti-hnRNPA1 antibody in A549 cells. IgG was employed as a negative control. **D** Immunoprecipitation of the hnRNPA1 protein by an anti-Flag antibody in HEK293 cells transfected with SRPK1-FLAG plasmid. The vector was used as a negative control. **E** Western blot analysis of SRPK1, hnRNPA1, PKM1, PKM2, PKM and PCNA protein levels in SRPK1-knockdown cells compared with control cells with quantification. **F** Western blot analysis of SRPK1, hnRNPA1, PKM1, PKM2, PKM and PCNA protein levels in SRPK1-overexpressing cells compared with control cells with quantification

**Fig. 9:**
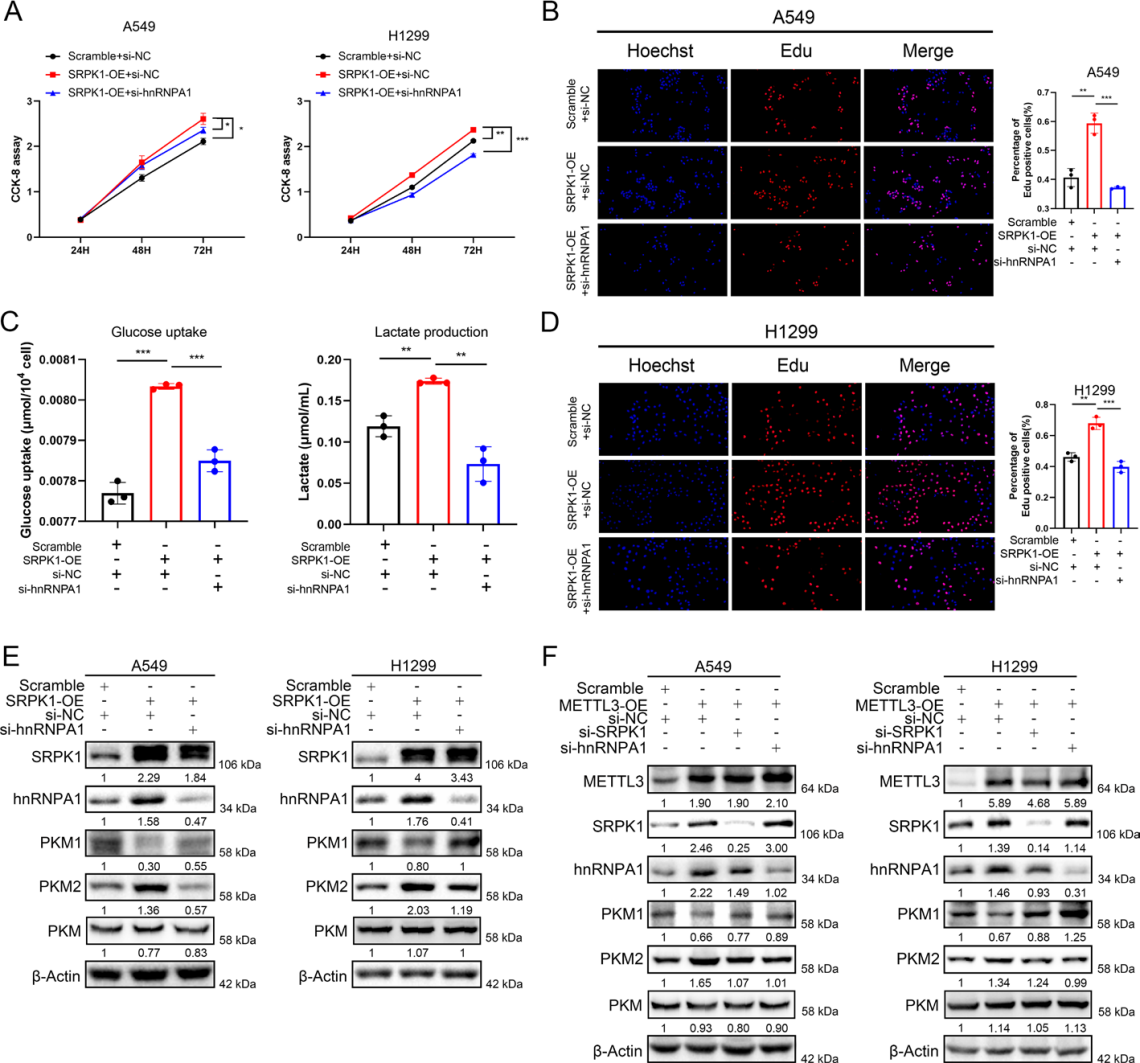
Inhibition of hnRNPA1 reversed the effects induced by SRPK1-expression. **A** hnRNPA1 knockdown suppress SRPK1-induced cell proliferation, as detected by CCK-8 assays. **B**, **D** hnRNPA1 knockdown suppress SRPK1-induced cell proliferation, as detected by EdU assays. **C** hnRNPA1 knockdown suppress SRPK1-induced high lactate production and glucose uptake. **E** hnRNPA1 knockdown suppress SRPK1-induced overexpression of hnRNPA1 and PKM2, and downregulation of PKM1. Total PKM level showed no significant difference. **F** Knockdown of SRPK1 and hnRNPA1 inhibits METTL3-induced overexpression of PKM2 and downregulation of PKM1. Total PKM level showed no significant difference. Data represent the mean (± SD) of three independent experiments, and two-way ANOVA and unpaired *t*-tests were carried out to validate the statistical significance. *P < 0.05; **P < 0.01; ***P < 0.001

Given the facts that PKM1/2 were two proteins evolved from the same pre-mRNA and SRPK1 is a protein kinase involved in the RNA splicing process, we aimed to find the RNA splicing factor which interacts with SRPK1 and mediates PKM splicing. To discover the unknown molecule interacting with SRPK1, we analysed A549 cells by immunoprecipitation (IP) assay using SRPK1-specific antibody and IgG antibody, the samples were separated by sodium dodecyl sulfate polyacrylamide gel electrophoresis (SDS-PAGE). Coomassie blue staining showed that SRPK1 was successfully pulled down and hnRNPA1 could be the splicing factor interacting with SRPK1 (Fig. 8A). To verify our hypothesis, further endogenous co-immunoprecipitation was performed using an anti-SRPK1 antibody, followed by western blot analysis and vice versa with the anti-hnRNPA1 antibody (Fig. 8B and 8C). Both assays indicated a bond between SRPK1 and hnRNPA1. For exogenous co-immunoprecipitation, the HEK293T cells were transiently transfected with Flag-tagged SRPK1 expression plasmids, and the anti-Flag antibody immunoprecipitation successfully confirmed the binding of SRPK1 and hnRNPA1 (Fig. 8D). In support of our findings, hnRNPA1 is known to be a key regulator of pre-PKM mRNA splicing and promotes the transition from pre-PKM to PKM2 variant. Subsequent western blot assays revealed that suppression of SRPK1 downregulated hnRNPA1 and PKM2 protein expression and upregulated PKM1 protein expression with stable total PKM levels, while SRPK1 overexpression in LUAD cells showed the opposite results (Fig. 8E and 8F), indicating that SRPK1 participated in PKM1/2 regulation through hnRNPA1. Furthermore, inhibition of hnRNPA1 antagonized the cell proliferation, glucose uptake and lactate production in A549 and H1299 cells induced by SRPK1 overexpression (Fig. 9A–D). The expression of PKM1 and PKM2 were also reversed by hnRNPA1 knockdown in SRPK1-overexpressing cells (Fig. 9E). Moreover, western blotting demonstrated that knockdown of SRPK1 or hnRNPA1, respectively, reversed the upregulation of PKM2 and downregulation of PKM1 induced by METTL3 overexpression (Fig. 9F). Collectively, these data showed that METTL3-regulated SRPK1 promotes LUAD malignant phenotype through mediating PKM splicing by interacting with hnRNPA1.

### Inhibition of METTL3 blocks SRPK1‑induced LUAD growth in vitro and in vivo

**Fig. 10:**
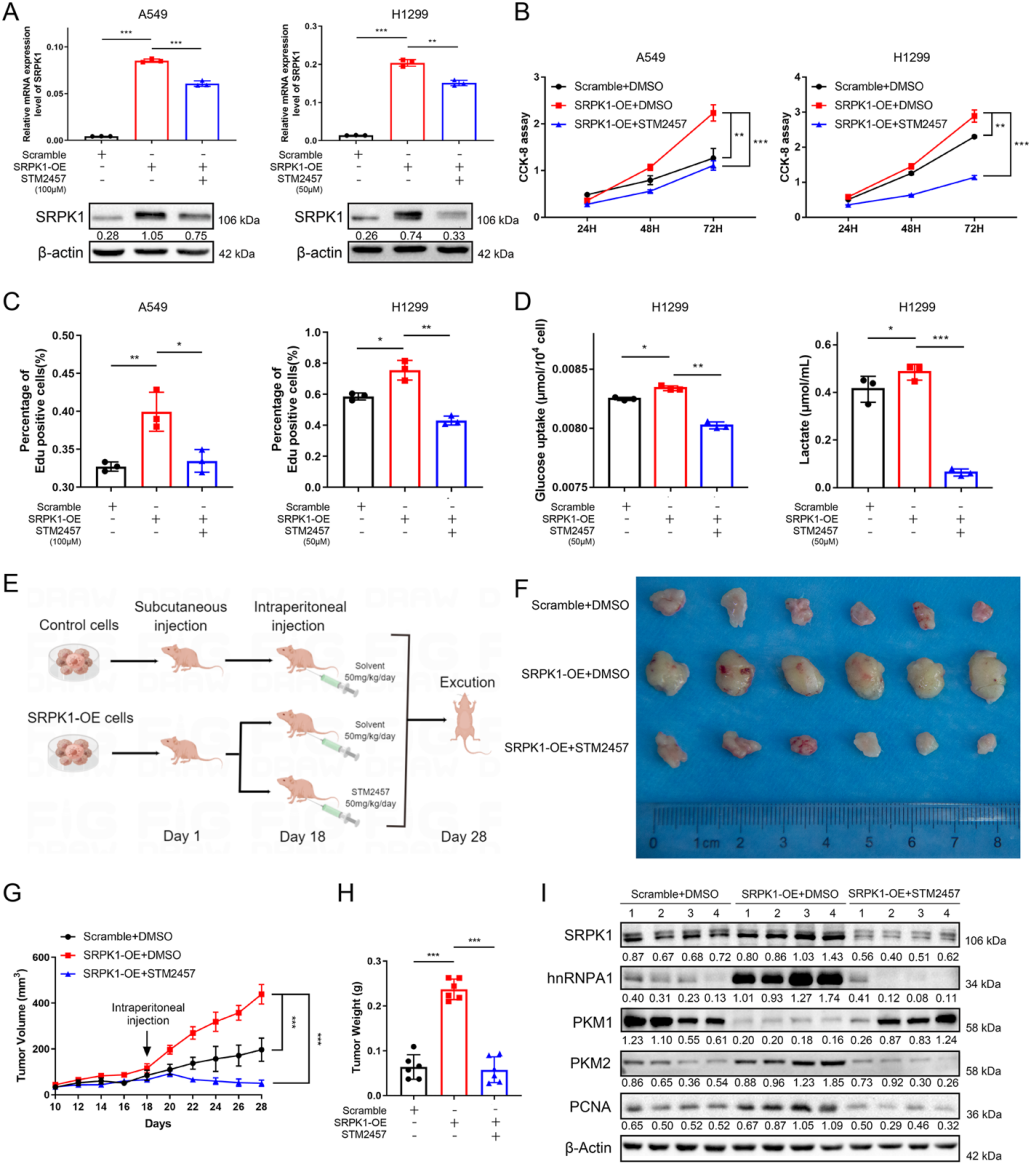
Inhibition of METTL3 blocks SRPK1‑induced cancer growth in vitro and in vivo. **A** The METTL3 inhibitor (STM2457) suppressed the mRNA and protein levels of SRPK1. **B** STM2457 inhibited SRPK1-induced hypercell viability, as detected by EdU assays. **C** Quantification of the EdU assay in SRPK1-overexpressing cells treated with STM2457. **D** Glucose uptake and lactate generation were measured in SRPK1-overexpressing cells treated with STM2457. **E** A diagram showing the procedure of the in vivo experiment. **F** Representative images of the xenograft tumours from three groups. **G**, **H** Tumour weight and volume in the SRPK1-overexpression and control groups without or with STM2457 treatment. **I** Western blot assay of SRPK1, hnRNPA1, PKM1, PKM2 and PCNA protein levels. Data are illustrated as the mean (± SD) of three separate experiments. Two-way ANOVA and unpaired *t*-tests were carried out to validate the statistical significance. *P < 0.05; **P < 0.01; ***P < 0.001

Next, we intended to explore the possibility of application of METTL3-specific inhibitors in LUAD, the catalytic inhibitory function of STM2457 in LUAD cells was examined (Fig. S5). SRPK1-overexpressing and control cells were treated with the METTL3 inhibitor STM2457 or DMSO solvent for 24 h, and then the cell lysate was collected for western blot analyses. Western blotting results showed that the expression of SRPK1 was significantly reduced by STM2457 (Fig. 10A). The CCK-8 assay and EdU assay demonstrated that STM2457 rescued the hyperactive cell proliferation induced by SRPK1 overexpression (Fig. 10B, C and Fig. S6). Glucose uptake analysis and lactate production analysis also confirmed the inhibitory function of STM2457 (Fig. 10D). To further determine the function of STM2457 in vivo, the SRPK1-overexpressing cells were injected into the flanks of BALB/c nude mice. Mice with established tumours were then randomly divided into three groups and treated with STM2457 or solvent, respectively (Fig. 10E, F). We then recorded the tumour volumes every other day (Fig. 10G), and weighed the tumours at the end of the experiment (Fig. 10H). The results showed that overexpression of SRPK1 significantly promoted tumour growth while application of STM2457 abrogated the additional growth. We further performed Western blot to examine the changes in protein levels of SRPK1 and other downstream molecular to verify our findings (Fig. 10I). These results reassured that regulation of SRPK1 was mediated by the methyltransferase catalytic function of METTL3, and suggested that STM2457 may be a promising clinical therapeutic strategy for patients with lung adenocarcinoma and extend the clinical usage of METTL3 inhibitors.

### The protein levels and correlations of relevant molecules in LUAD samples

**Fig. 11:**
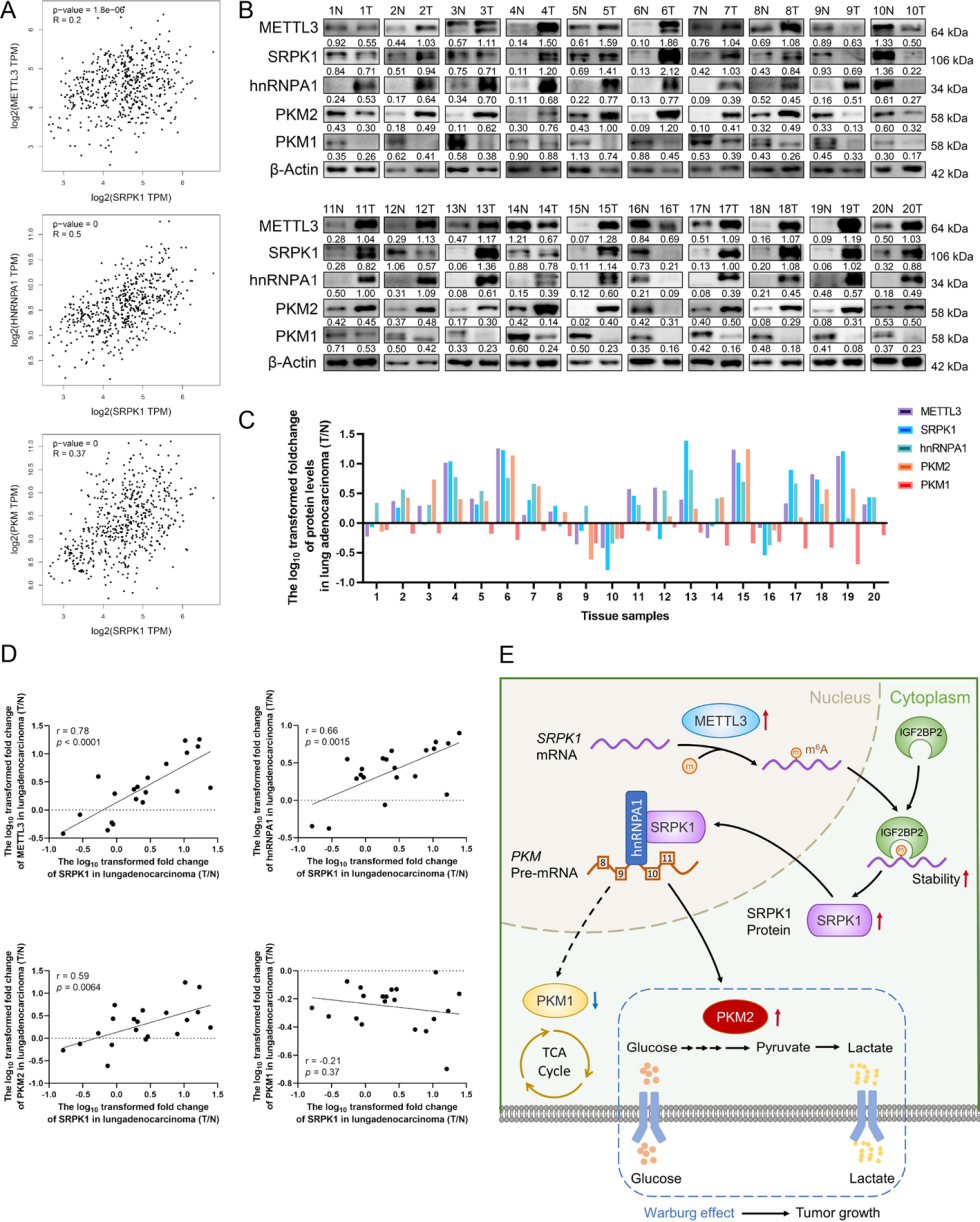
The correlations of relevant molecules in LUAD samples. **A** The correlation between SRPK1 and METTL3, SRPK1 and hnRNPA1, SRPK1 and PKM in LUAD was analysed by GEPIA. **B** Western blot analysis of protein levels of METTL3, SRPK1, hnRNPA1, PKM1 and PKM2 in 20 paired LUAD and adjacent tissues. **C** Relative quantification of protein levels in 20 paired LUAD and adjacent tissues. **D** The correlation between SRPK1 and METTL3, SRPK1 and hnRNPA1, SRPK1 and PKM1, SRPK1 and PKM2 in 20 paired LUAD tissues. **E** A diagram showing that METTL3 stabilized SRPK1 levels in an m^6^A-IGF2BP2-dependent manner and thus promoted glycolysis in LUAD

To find out the correlation between SRPK1 and relevant molecules in LUAD, we used the GEPIA database and revealed that SRPK1 is positively related to METTL3, hnRNPA1 and PKM (Fig. 11A). We further performed Western blot analyses of 20 paired human LUAD tissue samples and adjacent tissues to investigate the clinical relevance of METTL3, SRPK1, hnRNPA1, PKM2 and PKM1 expression (Fig. 11B). Among these, 65% of the tissue samples (13/20) showed consistently upregulated or downregulated METTL3, SRPK1, hnRNPA1 and PKM2 expression (Fig. 11C). Pearson correlation analysis revealed that SRPK1 levels were positively linked with METTL3, hnRNPA1 and PKM2, while negatively correlated with PKM1 (Fig. 11D). These results revealed a critical role of METTL3-mediated SRPK1 expression in LUAD progression.

## Discussion

Multiple epigenetic alterations, including chromatin remodelling, non-coding RNA expression, DNA methylation and post-transcriptional modulators, can contribute to LUAD tumour pathogenesis [37–40]. M^6^A modification is the most frequent post-transcriptional modification and essentially regulates the malignant tumour phenotype [41]. However, the possible association of RNA m^6^A modification in LUAD glycolytic metabolism is still poorly understood. In the present study, we demonstrated that METTL3-mediated m^6^A modification of SRPK1 promotes LUAD tumorigenesis by activating glycolysis via hnRNPA1-induced PKM splicing (Fig. 11E).

Accumulating evidence has revealed that abnormal levels of m^6^A and its linked proteins, such as erasers, writers, and readers, indicate a strong association with tumour progression and pathogenesis [42]. In the present study, we indicated that the levels of m^6^A RNA methylation and RNA methyltransferase METTL3 expression are markedly enhanced in LUAD. METTL3, the key component of the methyltransferase complex, can play different roles in multiple types of cancer [43, 44]. For example, METTL3 is upregulated and promotes tumour progression in colorectal cancer, gastric cancer, and breast cancer [23, 45, 46]. In contrast, METTL3 can act as a tumour suppressor in papillary thyroid cancer or endometrial cancer [47, 48]. This research revealed that METTL3 has an oncogenic activity in LUAD tumorigenesis, consistent with previous studies [49]. We first performed a series of gain and loss-of-function analyses in LUAD cells and indicated that METTL3 downregulation promoted cell proliferation and motility, while METTL3 overexpression exerted the opposite effects. In addition, its upregulation in xenografts resulted in a significant increase in tumour growth, which further indicated that METTL3 facilitated LUAD tumorigenesis. However, the functional mechanisms of METTL3 in LUAD might be complex due to the double-sided effects of METTL3 in different cancers, but in depth exploration of novel regulatory mechanisms of METTL3 in LUAD is urgently needed.

We further performed epitranscriptomic microarray analysis to identify candidate genes with high m^6^A methylation levels and increased mRNA levels. The results revealed top ten mRNAs, including SRPK1, were significantly hypermethylated in LUAD. Western blot, qRT‒PCR, MeRIP, luciferase reporter assay and rescue experiments were performed to identify SRPK1 as an essential METTL3 target in LUAD. SRPK1 is a protein kinase that participates in precursor mRNA processing and splicing, as well as other biological processes [14]. SRPK1 is overexpressed and acts as an oncogene in multiple cancers, including lung cancer [17, 50]. However, its epigenetic regulatory mechanism in LUAD remains unclear. This investigation indicated that SRPK1 was increased in LUAD due to METTL3-mediated m^6^A methylation. Moreover, m^6^A modifications containing mRNA transcripts can be recognized and lead to different fates by m^6^A readers. YTHDF2 and YTHDF3 reduces genes expression by mediating their mRNA decay [35, 51], while IGF2BPs modulates genes expression by promoting mRNA stability [36]. Much research has indicated that METTL3 increases targeted mRNA stability in an IGF2BP2-dependent manner in cancers [12, 23]. IGF2BP2 was also found to promote tumorigenesis in lung cancer [52]. Since we found that SRPK1 expression was upregulated after m^6^A modification, we further validated that METTL3 overexpression increased the stability of the SRPK1 transcript. Subsequently, our western blot results confirmed that the METTL3-induced overexpression of SRPK1 can be reversed by IGF2BP2. Collectively, we illustrated that METTL3-induced aberrant expression of SRPK1 promoted LUAD malignant phenotype by stabilizing SRPK1 expression in an IGF2BP2-dependent manner.

Regarding the exact mechanism by which METTL3 and its target SRPK1 promote LUAD progression, we considered aerobic glycolysis because cancer cells prefer glycolysis over OXPHOS to provide sufficient biomass and energy for rapid proliferation [26]. Previous literature has reported that METTL3 is essentially involved in glucose metabolism in various cancers [23, 53, 54], but the mechanism and function of SRPK1 in glucose metabolism in LUAD remains largely undetermined. Our data showed that SRPK1 overexpression accelerated the glycolytic rate as well as lactate production and glucose consumption in LUAD cells, and this phenomenon could be reversed by METTL3 knockdown. Interestingly, the quantification analysis of energy metabolites indicated that lactate and pyruvic acid were the two most significantly differentially expressed metabolites in SRPK1-overexpression cells, which led us to focus the impact of SRPK1 on the upstream kinase PKM that catalyses the production of pyruvic acid. In glycolysis, PKM is the last rate-limiting enzyme and has two isoforms resulting from the alternative *PKM* pre-mRNA splicing, due to the inclusion of either exon 9 (*PKM1*) or exon 10 (*PKM2*) [28]. PKM2 is known to facilitate aerobic glycolysis, whereas PKM1 normally promotes OXPHOS [55, 56]. Our western blot results confirmed that SRPK1 promoted glycolysis by increasing PKM2 expression and decreasing PKM1 expression. Since SRPK1 is a protein kinase, we were excited to find that RNA splicing factor hnRNPA1 interacted with SRPK1 to modulate PKM splicing. HnRNPA1 is an RNA-binding protein that is essentially linked with PKM alternative splicing [28]. Some research has reported that increased hnRNPA1 expression enhances glycolysis-dominant metabolism by downregulating PKM1/PKM2 ratio [30, 57]. We used co-IP and western blot assays to prove that SRPK1 interacted with hnRNPA1 to mediate PKM splicing in LUAD. In conclusion, these results showed that METTL3-induced SRPK1 upregulation promoted LUAD proliferation by accelerating glycolysis via interacting with hnRNPA1 and regulating PKM splicing.

With all these data unveiling the importance of METTL3 as a possible treatment target, we further applied a new selective METTL3 suppressor called STM2457. Research has elucidated its therapeutic impact in acute myeloid leukaemia [58], but little is known about its function and effects in LUAD. We demonstrated that STM2457 also had an anti-tumour effect in SRPK1-overexpressing LUAD xenograft tumours, which was also one of the first research where STM2457 was applied in LUAD xenograft models. According to the study by Ma et al. [59], molecular compounds can reverse disease progression by altering the level of a certain metabolic enzyme, which reveals the importance of cell metabolism in severe diseases. We here also examined the expression METTL3/SRPK1/hnRNPA1 signalling axis and PKM1/PKM2 ratio by western blot. Because STM2457 only affects the catalytic activity but not the protein expression of METTL3, these data also proved that METTL3 mediated SRPK1 expression by m^6^A modification rather than other regulation methods. And all the above results suggested that targeting METTL3 might be a potential therapeutic avenue for LUAD treatment.

Of course, our study also has limitations. For example, the question regarding the exact mechanisms by which SRPK1 activates hnRNPA1 in LUAD cells still needs further exploration. This mechanism might be dependent on certain specific protein domains in SRPK1, and we hope to answer these questions in our follow-up investigations.

## Conclusions

The current study validated the increased levels of m^6^A and METTL3 in LUAD and identified SRPK1 as a crucial downstream target of METTL3-mediated m^6^A modification in LUAD. Furthermore, it indicated a novel pathway by which METTL3-induced SRPK1 upregulation promotes LUAD proliferation by enhancing aerobic glycolysis via hnRNPA1-mediated PKM splicing. We are also one of the first to validate the effects of METTL3 inhibitor STM2457 in LUAD in vivo, which could pose a potential therapeutic strategy against LUAD.

### Supplementary Information

Supplementary Material 1.

## Data Availability

The datasets used and/or analysed during the current study are available from the corresponding authors upon reasonable request.

## Abbreviations

LUAD: Lung adenocarcinoma
m^6^A: *N*^6^-methyladenosine
NSCLC: Non-small-cell lung cancer
MTC: Methyltransferase complex
SRPK1: Serine–arginine protein kinase 1
SRSFs: Serine/arginine-rich splicing factors
hnRNPA1: Heterogeneous nuclear ribonucleoprotein A1
OXPHOS: Oxidative phosphorylation
ECAR: Extracellular acidification rate
HK: Hexokinase
PKM 1/2: Pyruvate kinase 1/2
LDH: Lactate dehydrogenase
RIP: RNA immunoprecipitation
G-6-P: Glucose-6-phosphate
F-6-P: Fructose-6-phosphate
F-1,6-P: Fructose-1,6-bisphosphate
G3P: Glyceraldehyde-3-phosphate
1,3-BPG: 1, 3-Diphosphoglyceric acid
PG: Phosphoglyceric acid
PEP: Phosphoenolpyruvic acid
