# Supplementary material for: N6-methyladenosine-modified SRPK1 promotes aerobic glycolysis of lung adenocarcinoma via PKM splicing
Source: Cell Mol Biol Lett. 2024 Aug 2;29:106. doi: 10.1186/s11658-024-00622-5 (PMC11295518; doi:10.1186/s11658-024-00622-5)
Supplement: Supplementary file 1 — Supplementary Material 1. [file 11658_2024_622_MOESM1_ESM.pdf]

## Supplemental Material

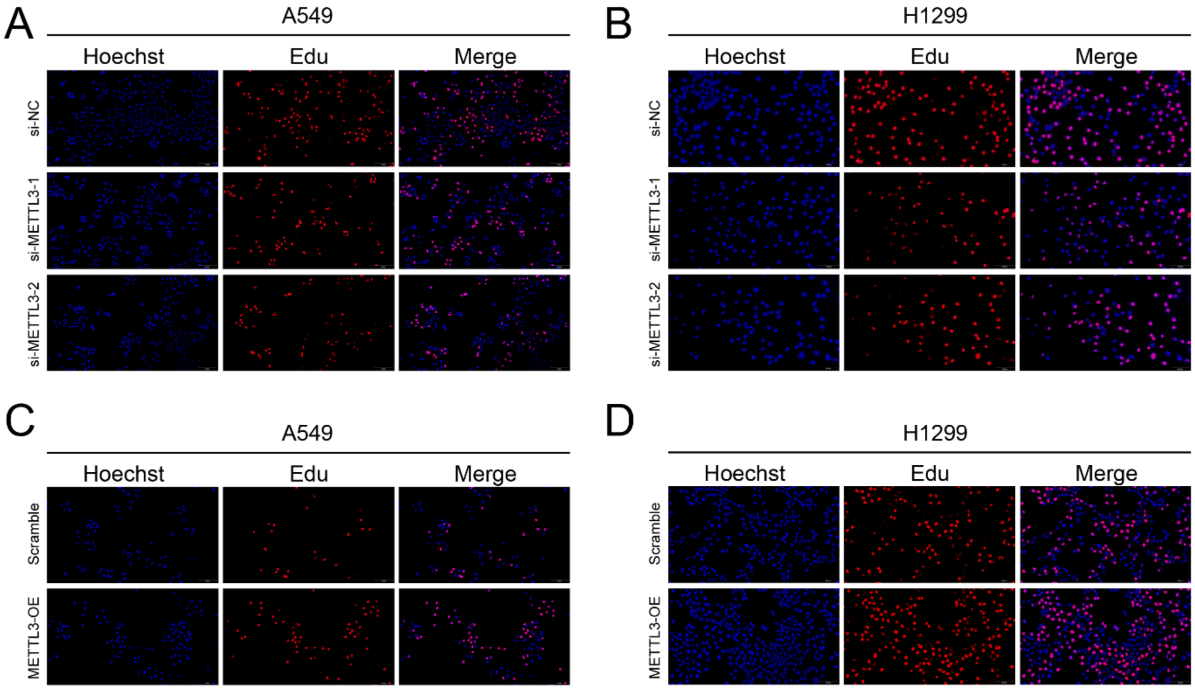


**Figure. S1 EdU analysis of LUAD cells with METTL3 alterations**

**(A, B)** Representative images of the EdU assay in A549 and H1299 cells with METTL3 knockdown.

**(C, D)** Representative images of the EdU assay in A549 and H1299 cells overexpressing METTL3.

A

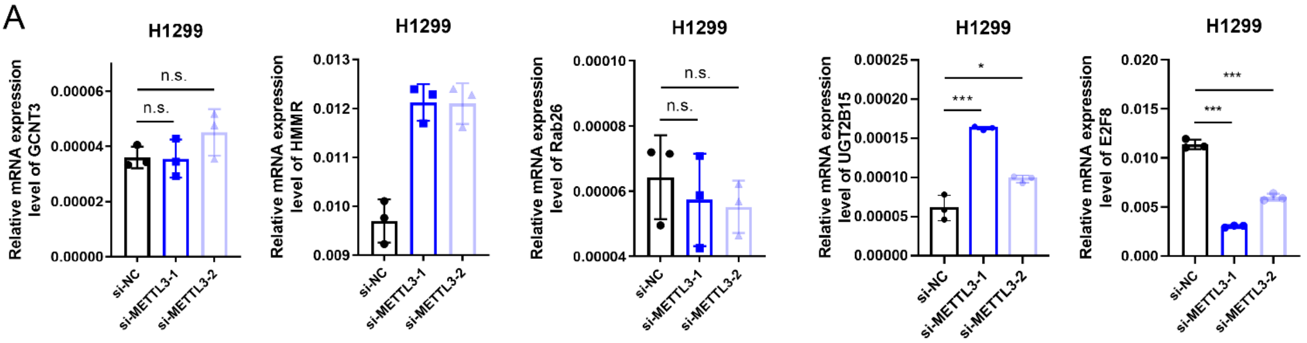


B

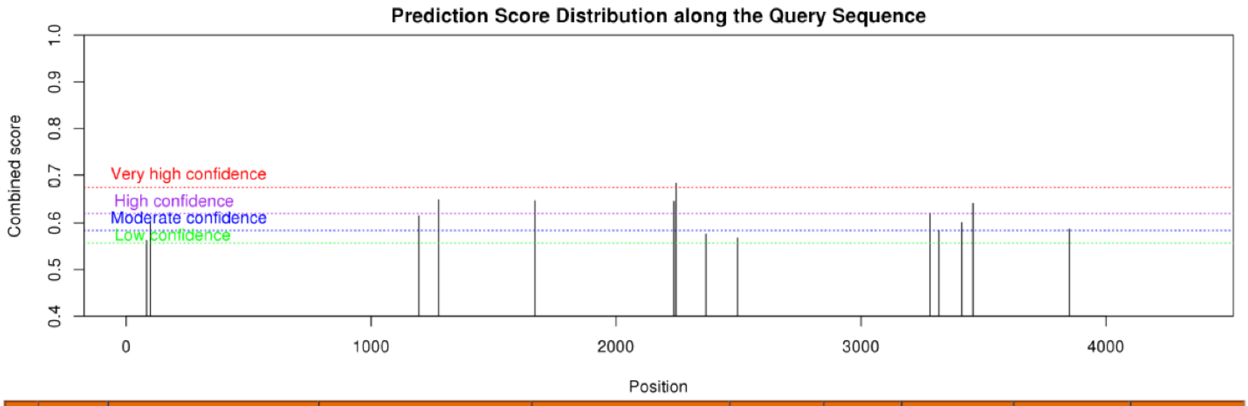


| # | Position | Sequence context | Structural context | Local structure visualization | Score(binary) | Score(knn) | Score(spectrum) | Score(combined) | Decision |
|---|---|---|---|---|---|---|---|---|---|
| 1 | 83 | GCUCC AGGCC CGAAA<br>GAAAA GGACC AAGGC<br>CAAGA AGGAC AAAGC | N/A | N/A | 0.58 | 0.809 | 0.506 | 0.562 | m$^6$A site (Low confidence) |
| 2 | 99 | AAAAG GACCA AGGCC<br>AAGAA GGACA AAGCC<br>CAAAG GAAAU CUGAA | N/A | N/A | 0.662 | 0.681 | 0.507 | 0.601 | m$^6$A site (Moderate confidence) |
| 3 | 1194 | GAGGA UCUAC AUAAU<br>GCUAA UGACU GUGAU<br>GUCCA AAAUU UGAAU | N/A | N/A | 0.654 | 0.722 | 0.55 | 0.615 | m$^6$A site (Moderate confidence) |
| 4 | 1275 | AGCAG CACAU CUCAA<br>GAAAC AGACU CUUGU<br>ACACC UAUAA CAUCU | N/A | N/A | 0.76 | 0.756 | 0.483 | 0.649 | m$^6$A site (High confidence) |
| 5 | 1669 | GCACG GCAUG CAUGG<br>CCUUU GAACU GGCCA<br>CAGGU GACUA UUUGU | N/A | N/A | 0.625 | 0.667 | 0.674 | 0.647 | m$^6$A site (High confidence) |
| 6 | 2237 | CCUUG GUUGG GCUCU<br>GCCAA AGACU AAUGG<br>ACUAA AAUGU GAAAC | N/A | N/A | 0.692 | 0.511 | 0.6 | 0.646 | m$^6$A site (High confidence) |
| 7 | 2245 | GGGCU CUGCC AAAGA<br>CUAAU GGACU AAAAU<br>GUGAA ACAGC CUCUU | N/A | N/A | 0.736 | 0.766 | 0.6 | 0.683 | m$^6$A site (Very high confidence) |
| 8 | 2367 | GAGCC CAUCC UUUUA<br>UUCAU UGACU CUAAG<br>AGUCA AAUUU UCUAG | N/A | N/A | 0.605 | 0.763 | 0.515 | 0.576 | m$^6$A site (Low confidence) |
| 9 | 2496 | CCGGG CUGCA UCAUC<br>UUCCU GGACU GUUUC<br>UGUUG UUCUC UGUGU | N/A | N/A | 0.72 | 0.623 | 0.352 | 0.568 | m$^6$A site (Low confidence) |
| 10 | 3281 | UUUUU GUUUU AAUUG<br>AGAAA AGACU UUGCA<br>AUUUU UUUUU UAGGA | N/A | N/A | 0.700 | 0.498 | 0.527 | 0.621 | m$^6$A site (High confidence) |
| 11 | 3318 | UUUUA GGAUG AGCCU<br>CUCCU AGACU UGACC<br>UAGAA UAUUA CAUAU | N/A | N/A | 0.570 | 0.676 | 0.593 | 0.584 | m$^6$A site (Moderate confidence) |
| 12 | 3411 | CACAG CCGCU UCUUC<br>AGCAU GGACC AAGUG<br>GGCCU UGGGG AUUGC | N/A | N/A | 0.579 | 0.697 | 0.620 | 0.601 | m$^6$A site (Moderate confidence) |
| 13 | 3457 | GCGUU CUCGA AGUGG<br>CUGUA GGACU CGAAU<br>UUACA GAAAG CCACA | N/A | N/A | 0.683 | 0.587 | 0.593 | 0.642 | m$^6$A site (High confidence) |
| 14 | 3851 | ACAUC UAAAG CAAUA<br>GACUA GAACU GAAUU<br>AUCUU CUACA UAGUA | N/A | N/A | 0.657 | 0.663 | 0.482 | 0.587 | m$^6$A site (Moderate confidence) |

**Figure. S2 Screening of potential targets of METTL3**

**(A)** The mRNA levels of different potential targets in METTL3-knockdown cells.

**(B)** The putative $m^6A$ modification sites in the sequence of SRPK1 predicted by the SRAMP database (http://www.cuilab.cn/sramp).

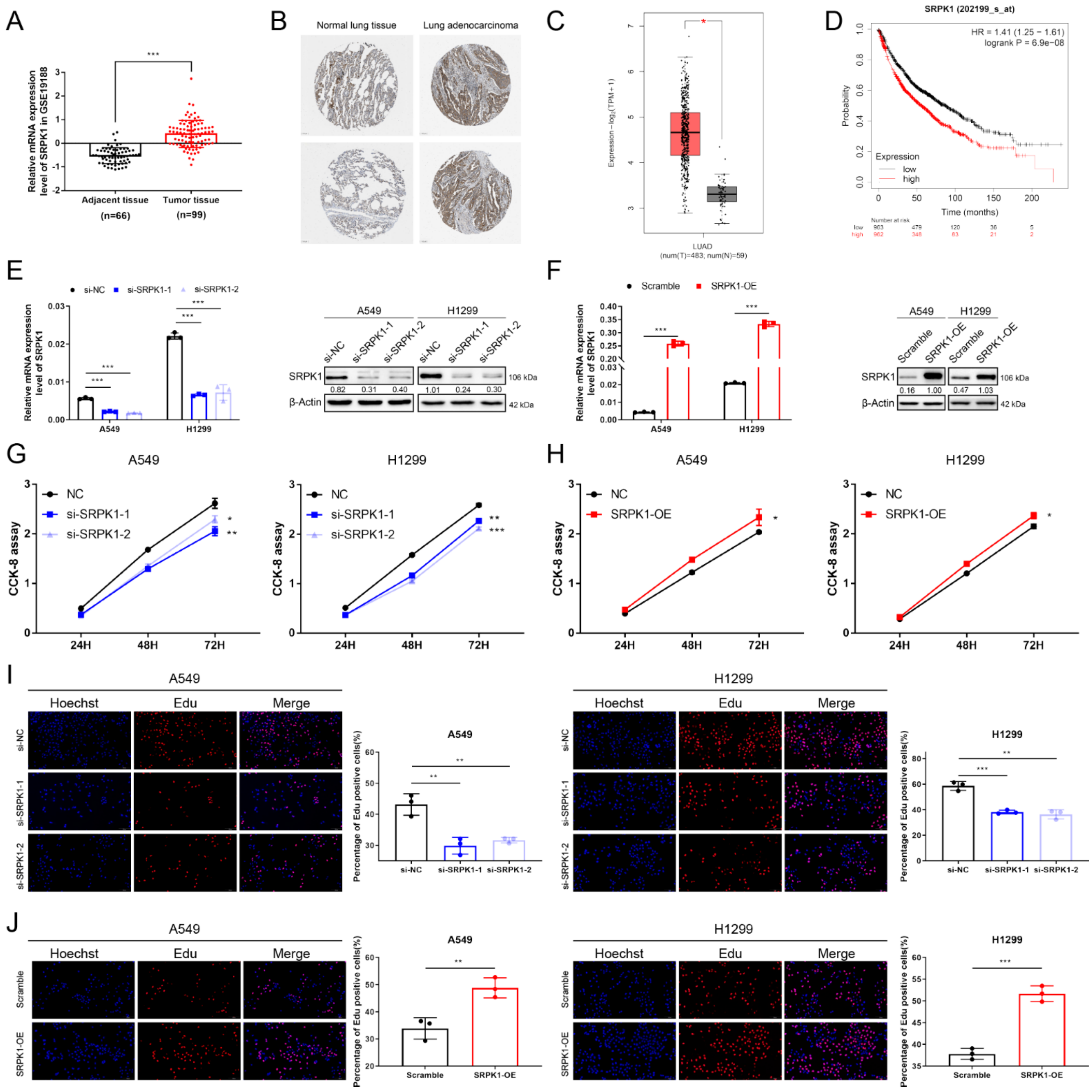


**Figure. S3 SRPK1 promotes cell proliferation in LUAD**

**(A)** Data from the GEO database (GSE19188) revealed that SRPK1 mRNA levels are significantly increased in lung cancer tissues compared to adjacent tissue samples.

**(B)** Representative immunohistochemistry images of SRPK1 in The Human Protein Atlas database (https://www.proteinatlas.org/).

**(C)** Data from the GEPIA database revealed that SRPK1 levels are significantly increased in LUAD samples compared to normal samples.

**(D)** Kaplan–Meier overall survival curves of SRPK1.

**(E)** The mRNA and protein levels of SRPK1 in SRPK1-knockdown cells.

**(F)** The mRNA and protein levels of SRPK1 in SRPK1-overexpressing cells.

**(G, H)** CCK-8 analysis of A549 and H1299 cell viability with SRPK1 knockdown or

overexpression.

**(I, J)** EdU analysis of A549 and H1299 cell viability with SRPK1 knockdown or overexpression.

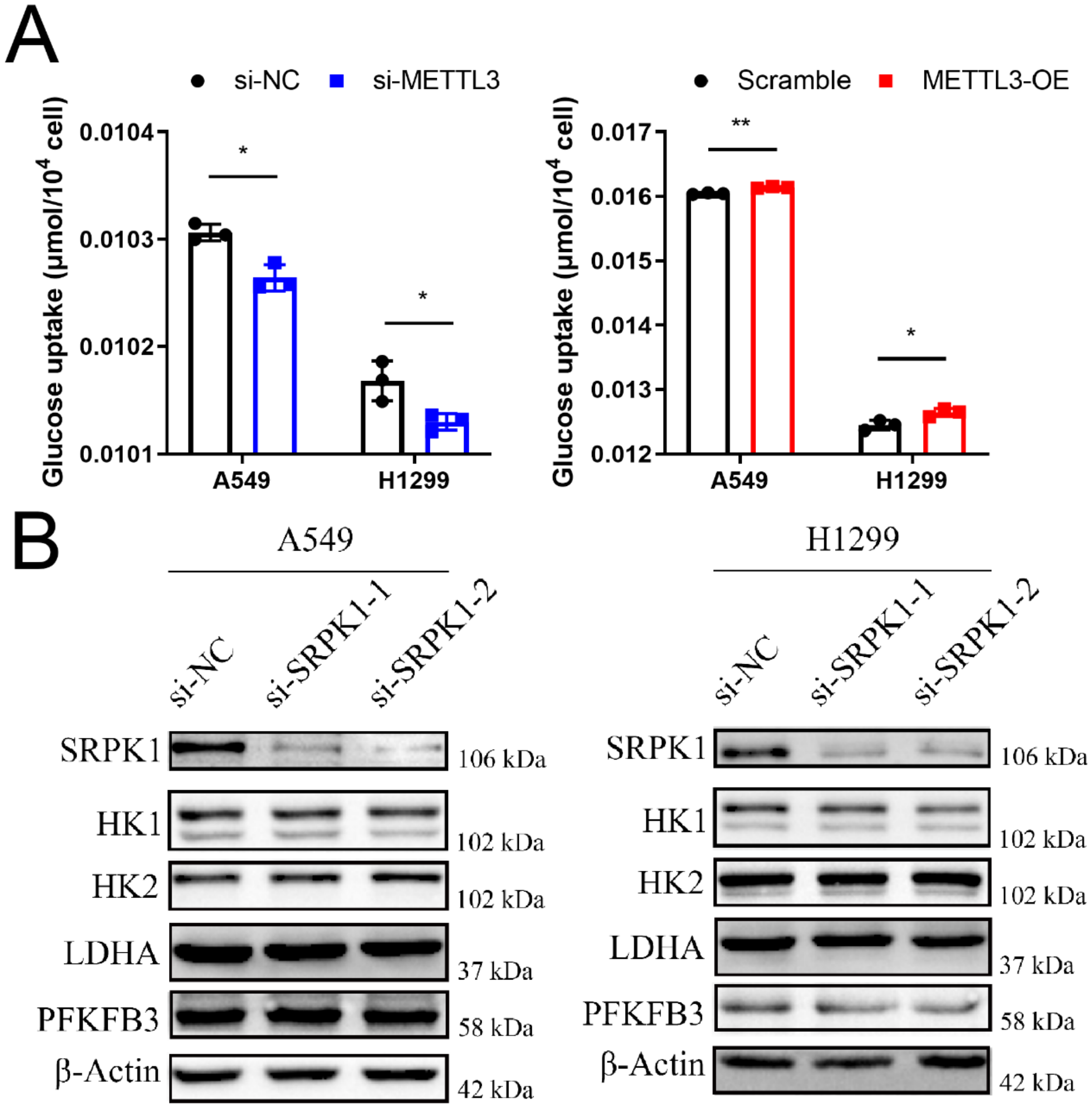


**Figure. S4 METTL3 and SRPK1 affects glycolysis in LUAD**

**(A)** Glucose uptake was measured in METTL3-knockdown and METTL3-overexpressing cells.

**(B)** Western blot analysis of SRPK1, HK1, HK2, LDHA and PFKFB3 protein levels in SRPK1-knockdown cells.

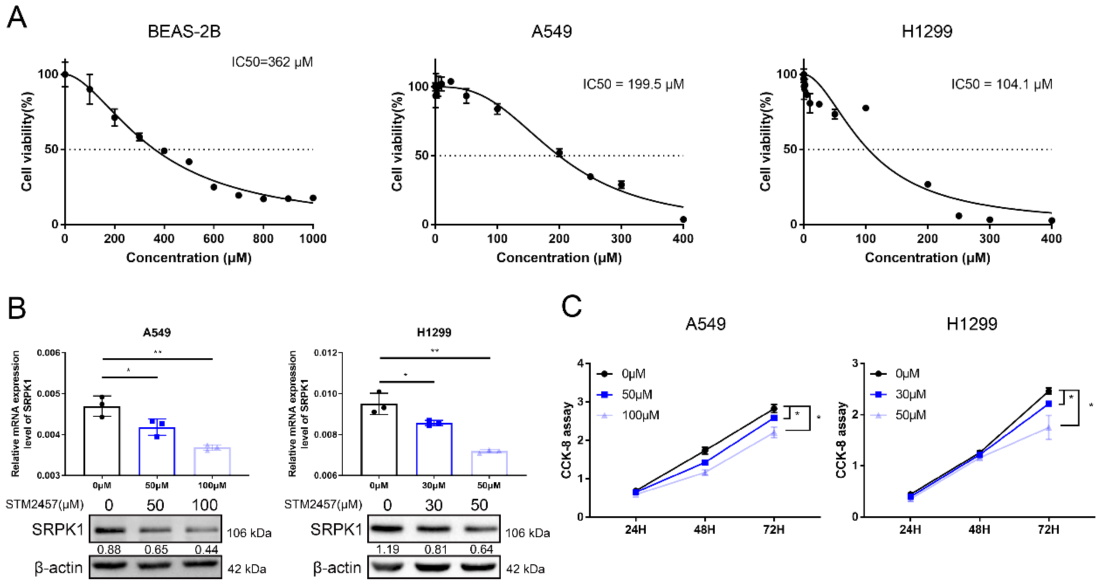


**Figure. S5 Inhibitory function of STM2457 in LUAD cells**

**(A)** The influence of STM2457 on cell viability in different cells.

**(B)** STM2457 inhibits the mRNA and protein levels of SRPK1 in a dose-dependent manner.

**(C)** STM2457 inhibits the viability of A549 and H1299 cells in a dose-dependent manner.

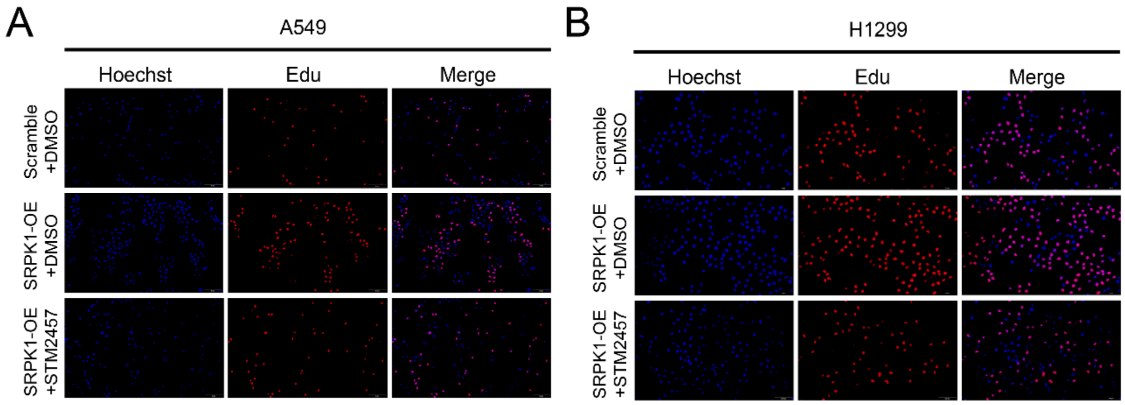


**Figure. S6 EdU analysis of LUAD cells treated with STM2457**

**(A, B)** Representative images of the EdU assay in A549 and H1299 cells with SRPK1 overexpression and treated with STM2457.

**Table S1. Clinicopathological features of 41 paired NSCLC patients.**

| Case | Sex | Age | Histology | TNM | Stage | Application |
|---|---|---|---|---|---|---|
| 1 | Male | 54 | Adenocarcinoma | T4N2M0 | IIIB | Epitranscriptomic microarray analysis |
| 2 | Male | 73 | Adenocarcinoma | T1cN0M0 | IA3 | |
| 3 | Female | 72 | Adenocarcinoma | T2aN0M0 | IB | |
| 4 | Male | 72 | Adenocarcinoma | T3N0M0 | IIB | |
| 5 | Male | 71 | Adenocarcinoma | T2bN0M0 | IIA | |
| 6 | Male | 46 | Adenocarcinoma | T4N2M0 | IIIB | |
| 7 | Female | 49 | Adenocarcinoma | T1bN0M0 | IA2 | $M^6A$ quantification |
| 8 | Female | 51 | Adenocarcinoma | T2N0M0 | IB | |
| 9 | Male | 54 | Adenocarcinoma | T1cN0M0 | IA | |
| 10 | Male | 65 | Adenocarcinoma | T1bN0M0 | IA2 | |
| 11 | Male | 65 | Adenocarcinoma | T1bN1M0 | IIB | |
| 12 | Female | 60 | Adenocarcinoma | T1bN0M0 | IA2 | |
| 13 | Male | 74 | Adenocarcinoma | T1bN0M0 | IA2 | |
| 14 | Male | 69 | Adenocarcinoma | T1bN0M0 | IA2 | |
| 15 | Female | 69 | Adenocarcinoma | T1bN0M0 | IA2 | |
| 16 | Female | 53 | Adenocarcinoma | T1cN0M0 | IA3 | |
| 17 | Female | 72 | Adenocarcinoma | T2N0M0 | IB | |
| 18 | Male | 56 | Adenocarcinoma | T1cN0M0 | IA3 | |
| 19 | Male | 69 | Adenocarcinoma | T2N0M0 | IB | |
| 20 | Male | 55 | Adenocarcinoma | T2N0M0 | IB | |
| 21 | Male | 74 | Adenocarcinoma | T1cN0M0 | IA3 | |
| 22 | Female | 61 | Adenocarcinoma | T1cN0M0 | IA3 | Western blotting |
| 23 | Female | 65 | Adenocarcinoma | T1aN0M0 | IA1 | |
| 24 | Male | 59 | Adenocarcinoma | T1cN0M0 | IA3 | |
| 25 | Male | 66 | Adenocarcinoma | T2bN0M0 | IIA | |
| 26 | Female | 63 | Adenocarcinoma | T1bN0M0 | IA2 | |
| 27 | Male | 77 | Mucinous adenocarcinoma | T2aN0M0 | IB | |
| 28 | Male | 55 | Adenocarcinoma | T3N0M0 | IIB | |
| 29 | Female | 56 | Adenocarcinoma | T1bN0M0 | IA2 | |
| 30 | Female | 67 | Adenocarcinoma | T1cN0M0 | IA3 | |
| 31 | Female | 75 | Adenocarcinoma | T1bN0M0 | IA2 | |

| | | | | | |
|---|---|---|---|---|---|
| 32 | Female | 65 | Adenocarcinoma | T1cN0M0 | IA3 |
| 33 | Female | 54 | Adenocarcinoma | T4N0M0 | IIIA |
| 34 | Male | 62 | Adenocarcinoma | T4N0M0 | IIIA |
| 35 | Male | 71 | Mucinous adenocarcinoma | T4N0M0 | IIIA |
| 36 | Male | 71 | Adenosquamous carcinoma | T2bN2M0 | IIIA |
| 37 | Female | 61 | Adenocarcinoma | T1bN0M0 | IA2 |
| 38 | Male | 70 | Adenocarcinoma | T1bN0M0 | IA2 |
| 39 | Male | 67 | Adenocarcinoma | T1bN1M0 | IIA |
| 40 | Male | 54 | Adenocarcinoma | T1cN1M0 | IIA |
| 41 | Female | 82 | Adenocarcinoma | T1cN0M0 | IA3 |

**Table S2. Sequences of siRNAs.**

| siRNA | Sense |
|---|---|
| si-NC | 5′-TTCTCCGAACGTGTCACGT-3′ |
| si-METTL3-1 | 5′-CTGCAAGTATGTTCACTATGA-3′ |
| si-METTL3-2 | 5′-CGTCAGTATCTTGGGCAAGTT-3′ |
| si-SRPK1-1 | 5′-GGCAATGAAAGTAGTTAAA-3′ |
| si-SRPK1-2 | 5′-CCATGTGATCCGAAAGTTA-3′ |
| si-IGF2BP2-1 | 5′-TGAAGCTGGAAGCGCATAT-3′ |
| si-IGF2BP2-2 | 5′-CCCGCATCAUCACTCTTAT-3′ |
| si-IGF2BP1 | 5′-GGAAAUAAUGAAGAAAGUUCGTT-3′ |
| si-IGF2BP3 | 5′-GGUGAAUGAACUUCAGAAUTT-3′ |
| si-YTHDF1 | 5′-GGACAGUCAAAUCAGAGUATT-3′ |
| si-YTHDF2 | 5′-GAAGTCTGTTGTGGACTATAATT-3′ |
| si-hnRNPA1 | 5′-CAGCUGAGGAAGCUCUUCATT-3′ |

**Table S3. Sequences of Primers for qRT-PCR.**

| Gene | Forward | Reverse |
|---|---|---|
| METTL3 | 5′-AAGCTGCACTTCAGACGAAT-3′ | 5′-GGAATCACCTCCGACACTC-3′ |
| SRPK1 | 5′-GGTGTGCCAGTCTTCCTCAAC-3′ | 5′-GGTCCGTTATGTTCTTGCTCTTG-3′ |
| HMMR | 5′-ATGATGGCTAAGCAAGAAGGC-3′ | 5′-TTTCCCTTGAGACTCTTCGAGA-3′ |
| E2F8 | 5′-CCAACCCTGCTGTGAATA-3′ | 5′-TTTCTGGCTCATTACCCT-3′ |

| | | |
|---|---|---|
| GCNT3 | 5′-TCAAAGAGGCGGTCAAAGCAA-3′ | 5′-GCATAAACCACCCGAACCAG-3′ |
| Rab26 | 5′-GTCTGCTGGTGCGATTCAAG-3′ | 5′-GCATGGGTAACACTGCGGA-3′ |
| UGT2B15 | 5′-CTTCTGAAAATTCTCGATAGATGGAT-3′ | 5′-CATCTTTACAGAGCTTGTTACTGTAGTCAT-3′ |
| PKM1 | 5′-CAGCCAAAGGGGACTATCCT-3′ | 5′-GAGGCTCGCACAAGTTCTTC-3′ |
| PKM2 | 5′-TCACCAAGTCTGGCAGGTCTG-3′ | 5′-CATTCATGGCAAAGTTCACCCGGA-3′ |
| β-actin | 5′- CACAGAGCCTCGCCTTTGCC-3′ | 5′-ACCCATGCCCACCATCACG-3′ |

**Table S4. Sequences of Primers for MeRIP-PCR.**

| Gene | Forward | Reverse |
|---|---|---|
| SRPK1-MeRIP | 5′-CTGCCAAAGACTAATGGACT-3′ | 5′-TGCTGGCAATAGGATATGCAC-3′ |
| E2F8-MeRIP | 5′-TGGACAATCAGTTGCTGTGAC-3′ | 5′-CTCCAAATCAGTCTGTGTACT-3′ |
| β-actin | 5′-CACAGAGCCTCGCCTTTGCC-3′ | 5′-CCCATGCCCACCATCACG-3′ |

**Table S5. Results of energy metabolites quantification.**

| Compounds | Class | Scramble | | | SRPK1-OE | | |
|---|---|---|---|---|---|---|---|
| | | NC1 | NC2 | NC3 | OE1 | OE2 | OE3 |
| ATP | Nucleotide and Its metabolomics | 0.12367444806355098 | 0.3886496424367185 | -1.7893860423529293 | -0.36402378425751647 | 1.0968209576905192 | 0.544264778419655 |
| Guanosine-diphosphate | Nucleotide and Its metabolomics | 0.4821898233518979 | 0.32617988320291497 | -1.9845100782618657 | -0.021857334727365483 | 0.5503038230075922 | 0.6476938834268292 |
| dUMP | Nucleotide and Its metabolomics | 1.1292899772000007 | 0.7357267016231717 | -1.740314768681397 | 0.27377464504151816 | -0.21284957879224645 | -0.1856269763910441 |
| dTMP | Nucleotide and Its metabolomics | 1.3102327481818414 | 0.6549894143860762 | -1.626436635839202 | 0.21093358814395377 | -0.1930232849203504 | -0.3566958299523242 |
| UMP | Nucleotide and Its metabolomics | -0.2890404524538688 | -0.4767044055713878 | -1.6370899630676135 | 0.8549943085565919 | 0.9742007915042474 | 0.5736397210320316 |
| IMP | Nucleotide and Its metabolomics | 1.2971820019870093 | 1.0109481917192482 | -1.3142580417445529 | -0.7216400806274622 | -0.06467077413476434 | -0.20756129719947877 |
| dAMP | Nucleotide and Its metabolomics | 1.3970114271772232 | 0.6168357995861715 | -1.5031389438869012 | 0.2856105156661253 | -0.32812082796782166 | -0.4681979705747983 |

| | | | | | | | |
|---|---|---|---|---|---|---|---|
| AMP | Nucleotide and Its metabolomics | 1.4147815143995055 | 0.8716234493441591 | -1.3018965246933838 | -0.6899499676935817 | -0.03701146247658846 | -0.2575470088801094 |
| UDP-GlcNAc | Nucleotide and Its metabolomics | 1.2217011496427956 | 1.1082473465277223 | 0.24833804951798275 | -0.8931654849128257 | -0.8433526360961394 | -0.8417684246795356 |
| Cyclic-AMP | Nucleotide and Its metabolomics | 0.7266616837527008 | 0.14686558681163572 | -1.988811526024269 | 0.563015399120985 | 0.3891282913675629 | 0.16314056497138438 |
| Guanosine | Nucleotide and Its metabolomics | 0.7306870773478912 | 1.0351870658136677 | 0.9588529116404317 | -0.903500994679005 | -0.8975938291249201 | -0.9236322309980651 |
| Adenine | Nucleotide and Its metabolomics | 1.2706655251487131 | 0.9191066528749292 | 0.4557551625150764 | -0.88259831635411 | -0.8811856814198405 | -0.8817433427647683 |
| Inosine | Nucleotide and Its metabolomics | 0.8013250393162923 | 1.23637077586528 | 0.6455442223724569 | -0.8583740525430448 | -0.8394117722734822 | -0.9854542127375017 |
| ADP | Nucleotide and Its metabolomics | -0.11426860811458162 | -0.311513159728053 | -1.5617715749656778 | -0.13904737352548235 | 1.3550385187707084 | 0.7715621975630849 |
| Uracil | Nucleotide and Its metabolomics | 0.7250139991054931 | 0.6768813399965828 | 1.24438259286641 | -0.6155111003839318 | -0.8995442203342217 | -1.1312226112503319 |
| NicotinaMide-adenine-dinucleotide(NAD) | Nucleotide and Its metabolomics | -0.7503339499245307 | -1.144855686907894 | 0.34978726185208536 | 0.9419607992680261 | -0.6817913564980935 | 1.285232932210405 |
| Tyrosine | Amino acids | -0.35577251947124505 | -0.44396593389357814 | -1.5273836266264909 | 1.3706026505186013 | 0.539100021919907 | 0.417419407552805 |
| L-Leucine | Amino acids | -0.568226478048916 | -0.6813516681388057 | -1.1637733175124765 | 1.5836270826406216 | 0.47149357882020854 | 0.35823080223936804 |
| Lysine | Amino acids | -0.2658090341707288 | -0.5484107530172198 | -1.1352534394763507 | 1.814097117196531 | 0.2085410549812133 | -0.0731649455134451 |
| Threonine | Amino acids | -0.5612585472472892 | -0.6289546298036599 | -1.2318839782287503 | 1.5324241280364528 | 0.5627008956107284 | 0.32697213163251865 |
| L-Glutamic-acid | Amino acids | 1.6846286198932954 | 0.7543250938948229 | -0.37236773506666887 | -0.5530284428547273 | -0.79610859022191 | -0.7174489456448137 |

| | | | | | | | |
|---|---|---|---|---|---|---|---|
| Serine | Amino acids | -0.6433651071095068 | -0.794323426903204 | -1.1468889408394045 | 1.386252410044135 | 0.6058768819740179 | 0.5924481828339606 |
| L-Alanine | Amino acids | -0.8327669013199143 | -0.7974868567430485 | -0.802252359727094 | 1.5745381123506799 | 0.728717497908979 | 0.1292505075303982 |
| Itaconic-acid | Amino acids | 1.1163332169820737 | 0.07733137013342084 | -1.8653807247470156 | 0.4203130374401589 | 0.29877660717057436 | -0.047373506979209355 |
| L-citrulline | Amino acids | 0.7697088934911738 | 1.4862088576725219 | -0.8942126934448058 | -0.29094840338183503 | 0.07339266002802923 | -1.144149314365082 |
| L-Cystine | Amino acids | 0.8880456419431144 | -0.38048264658686837 | -0.22241327657393947 | -1.7027813528338394 | 0.946340728756393 | 0.47129090529514384 |
| L-Asparagine | Amino acids | -0.8141907932934663 | -0.769761961091288 | -1.1046039167077657 | 1.1727667829159623 | 0.7683506340871652 | 0.7474392540893936 |
| cis-Aconitic-acid | Amino acids | 1.0694546298474974 | 0.1664853522592422 | -1.8968765343589322 | 0.4407586666507239 | 0.18722999120437098 | 0.03294789439709614 |
| Malic-acid | Amino acids | 1.5754412399700084 | 0.576699202389945 | -1.347312717273879 | 0.050849797726006445 | -0.4549833537307724 | -0.4006941690813123 |
| Arginine | Amino acids | -0.38918249360995383 | -0.5449511069358074 | -1.2709947025830874 | 1.6613461774585128 | 0.39067052711123657 | 0.15311159855909842 |
| Citric-acid | Organic acid And Its derivatives | 1.0142947021158644 | 0.8353501223522074 | -1.6108625949574167 | 0.4468948569223084 | 0.006250688885923985 | -0.6919277753188845 |
| Succinic Acid | Organic acid And Its derivatives | 0.7336057183651052 | -0.02309407768239212 | -1.9364718805557495 | 0.783061998053887 | 0.2285360904841921 | 0.21436215133496056 |
| Alpha-Ketoglutaric-Acid | Organic acid And Its derivatives | 0.44732026748413 | 0.13395941205121223 | -1.9605338275189852 | 0.9050168565227851 | 0.1789785112357093 | 0.2952587802251507 |
| Lactate | Organic acid And Its derivatives | -0.9416455736276141 | -0.8993023114133198 | -0.8887808976201061 | 1.009069532631388 | 0.9499734573139611 | 0.7706857927156902 |
| Argininosuccinic-acid | Organic acid And Its derivatives | 0.6430292942883963 | 1.3859055109530027 | -0.08939294400557483 | -1.195359480383898 | 0.31783639549527054 | -1.0620187763471935 |

| | | | | | | | |
|---|---|---|---|---|---|---|---|
| Fumaric-acid | Organic acid And Its derivatives | 0.8228722509291291 | -0.3153890824004085 | -1.892515814064719 | 0.48678750252711384 | 0.4756804119879864 | 0.4225647310208995 |
| Isocitric-acid | Organic acid And Its derivatives | 1.0937818982598497 | 0.8714100763238785 | -1.2849227424491771 | 0.5220269841912366 | -0.15506015958227878 | -1.047236056743511 |
| Pyruvic-acid | Organic acid And Its derivatives | -0.9127378722050695 | -0.9127378722050695 | -0.9127378722050695 | 0.9048821912227614 | 0.8905420426838682 | 0.9427893827085794 |
| D-Fructose-6-phosphate | Phosphate sugars | 0.19929276015651606 | 0.23290441952670354 | -1.7527301895987586 | 1.34489193843954 | 0.09949820764277967 | -0.12385713616678123 |
| D-Glucose-1-phosphate | Phosphate sugars | 0.04577044465630786 | 0.06314282164585126 | -1.7203535922999391 | 1.3522895345448458 | 0.4228628113586436 | -0.16371201990570927 |
| Sedoheptulose-7-phosphate | Phosphate sugars | 0.22598298811568904 | -0.37093518453061164 | -1.8517486184737395 | 0.8082627573546591 | 0.70118780625835 | 0.4872502512756546 |
| D-Ribulose-5-phosphate | Phosphate sugars | 1.406138439156883 | 0.7102688557364485 | 0.16144959095695388 | -1.44197687801965 | -0.2396398895191813 | -0.5962401183114494 |
| D-Glucose-6-phosphate | Phosphate sugars | -0.5370869466766898 | -0.3823962192813952 | -1.475641647598052 | 1.3300031335762417 | 0.6932500418030977 | 0.3718716381767952 |
| D-Erythrose 4-phosphate | Phosphate sugars | 0.8506226450259701 | 0.6625136220089227 | 0.2184675792580587 | -1.2591476110059756 | -1.2591476110059756 | 0.7866913757189999 |
| 6-Phosphogluconic-acid | Phosphate sugars | -0.8806245914831273 | -0.8130340289200075 | -1.0004225985210593 | 0.8401207248829515 | 1.1898899577113524 | 0.66407053632989 |
| Fructose-1,6-bisphosphate | Phosphate sugars | -0.7083390610781175 | -0.7259236675105872 | -0.8644058848084295 | 0.03903276692923692 | 0.5512021467501006 | 1.7084336997177951 |
| 2-Phospho-D-glyceric acid | Phosphoric acids | -0.8704251519586399 | -0.9605844575509531 | -0.889029532928899 | 0.7052894967143236 | 0.9740378862813905 | 1.0407117594427773 |
| Glyceraldehyde-3-phosphate | Phosphoric acids | -0.6600310517973051 | -0.9116893222477357 | -1.0401721130277741 | 0.3932304352270472 | 0.976359712657482 | 1.2423023391882864 |
| Dihydroxyacetone-phosphate | Phosphoric acids | -0.7033277505530542 | -0.8798821476795112 | -1.0522187777809646 | 0.4540881674027892 | 0.9516489355199128 | 1.2296915730908289 |

| | | | | | | | |
|---|---|---|---|---|---|---|---|
| Phosphoenolpyruvic-acid | Phosphoric acids | -0.8521210982528727 | -0.9344113678342421 | -0.9272621338176 | 0.6659538484951449 | 1.0061782666117385 | 1.0416624847978315 |
| Glycerol-3-phosphate | Phosphoric acids | -0.7108086987627604 | -0.8060228213217904 | -1.1571601795585114 | 0.6231553154535444 | 0.9394791451487076 | 1.111357239040811 |
| BPG | Phosphoric acids | -0.8902477252001455 | -0.9051504979945294 | -0.9183180527326454 | 0.9168017425879121 | 0.6864700471184754 | 1.1104444862209326 |
| 3-phosphoglycerate | Phosphoric acids | -0.8668032777096543 | -0.9035619908698762 | -0.9494045085262286 | 0.7009717799566342 | 1.038765413532014 | 0.9800325836171104 |
| Flavin-mononucleotide | CoEnzyme and vitamins | 1.528234572910535 | 0.9686265622207326 | -0.9115898450383495 | -0.3345690361551988 | -0.6498574892865696 | -0.6008447646511492 |
| Succinyl-CoA | CoEnzyme and vitamins | -0.8911178118719895 | -0.8531776710452779 | -0.9442354301926903 | 0.7489079214744565 | 1.2385907822921194 | 0.701032209343382 |
| Acetyl-CoA | CoEnzyme and vitamins | 0.6976321400620248 | 0.08690565471265178 | -1.9919393587322523 | 0.5540956426120612 | 0.4202160433244122 | 0.2330898780210994 |
| Glutamine | Amino acid derivatives | -0.35474224447541153 | -0.45528303645999635 | -1.231476678099395 | 1.7636337337805896 | 0.11313521961050285 | 0.16473300564370869 |
| L-Aspartate | Amino Acid metabolomics | 1.3485155755647644 | 0.8431427046507263 | 0.4223715561778996 | -0.7758680043048545 | -0.9394250799225737 | -0.8987367521659604 |
| Xyluose-5-phosphate | Carbohydrate metabolomics | 1.3550729114705178 | 0.7283799980390798 | 0.3751512655180455 | -1.0062740618570034 | -0.2650345736098925 | -1.187295539560746 |
| Phosphorylethanolamine | LPE | 1.0235515456296898 | 1.0913727005350902 | 0.5772158057741658 | -0.81200981699051 | -0.965214495773758 | -0.9149157391746778 |
| 3-phenyllactic-acid | Organic Acid And Its Derivatives | 1.0580238570443277 | 1.143812968292269 | 0.4546543907161229 | -0.8386269234333324 | -0.9794884592677574 | -0.8383758333516311 |
